# Transmembrane effector substrates of type IV secretion systems: mechanisms of secretion and insertion into host cell membranes

**DOI:** 10.1093/femsml/uqag007

**Published:** 2026-02-27

**Authors:** Sarah-Maria Trenz, Ann-Kathrin Kuwertz, Josua Carl, Samuel Wagner

**Affiliations:** Section of Cellular and Molecular Microbiology, Interfaculty Institute of Microbiology and Infection Medicine (IMIT), University of Tübingen, Elfriede-Aulhorn-Str. 6, 72076 Tübingen, Germany; Section of Cellular and Molecular Microbiology, Interfaculty Institute of Microbiology and Infection Medicine (IMIT), University of Tübingen, Elfriede-Aulhorn-Str. 6, 72076 Tübingen, Germany; Quantitative Biology Center (QBiC), M3 Research Center, University of Tübingen, Otfried-Müller-Straße 37, 72076 Tübingen, Germany; Section of Cellular and Molecular Microbiology, Interfaculty Institute of Microbiology and Infection Medicine (IMIT), University of Tübingen, Elfriede-Aulhorn-Str. 6, 72076 Tübingen, Germany; German Center for Infection Research (DZIF), partner-site Tübingen, 72076 Tübingen, Germany; Excellence Cluster “Controlling Microbes to Fight Infections” (CMFI), Elfriede-Aulhorn-Str. 6, 72076 Tübingen, Germany

**Keywords:** membrane effector proteins, topology, hydrophobicity, type IV secretion systems A and B, protein secretion, pathogen-containing vacuole, eukaryotic membrane protein biogenesis

## Abstract

Intracellular Gram-negative pathogens employ either type IVA or type IVB secretion systems (T4SSs) to translocate effector proteins into host cells, where they modulate cellular processes to facilitate infection and promote intracellular survival. Roughly one-third of these effectors harbor hydrophobic transmembrane domains and are thus destined for integration into host cell membranes during infection. Many of these transmembrane domain-containing effectors (TMEs) localize to the membrane of the pathogen-containing vacuole, thereby contributing to its formation and remodeling. Despite the biological relevance of TMEs, the detailed molecular mechanisms governing their translocation via T4SSs and subsequent membrane integration in the host cell remain insufficiently understood. In this review, the biophysical characteristics of T4SS-secreted TMEs are systematically examined, including predictions of membrane topology and hydrophobicity. These analyses are then contextualized through comparison with recent structural analysis of both T4ASS and T4BSS machineries, as well as with mechanistic principles of eukaryotic membrane protein biogenesis. This integrative approach enables the conceptual reconstruction of the potential pathways by which TMEs are translocated through the T4SS and subsequently targeted and inserted into host membranes, offering new mechanistic insights into the poorly understood handling of bacterial TMEs from both the pathogen and host perspectives.

## Teaser

In this review, the biophysical characteristics of T4SS-secreted transmembrane domain-containing effectors (TMEs) are systematically examined and analysed in context of the T4ASS and T4BSS structures, as well as mechanistic principles of eukaryotic membrane protein biogenesis, to contribute to the understanding of TME handling from the pathogen and host perspectives.

## Introduction

Many intracellular Gram-negative pathogens rely on type IV secretion systems (T4SS) to translocate effector proteins into host cells and thereby manipulate key cellular processes to evade immune responses, acquire nutrients, and survive intracellularly. Since intracellular bacterial replication often occurs within the protected environment of a membrane-bound compartment known as the pathogen-containing vacuole (PCV) (Costa et al. 2024), T4SS-secreted effectors must traverse not only the bacterial envelope but also the PCV membrane to reach the host cytosol.

T4SSs are classified into two major subtypes based on their structural and functional features: the type IVA secretion systems (T4ASS), exemplified by the VirB/D4 system of *Brucella* spp., *Bartonella* spp., and *Rickettsia* spp. (Dehio 2008, Gillespie et al. 2016, Sankarasubramanian et al. 2016), and the more complex type IVB secretion systems (T4BSS), including the Dot/Icm (defective in organelle trafficking/intracellular multiplication) system of *Legionella pneumophila, Coxiella burnetii*, and *Rickettsiella grylii* (Berger and Isberg 1993, Brand et al. 1994, Seshadri et al. 2003, Zusman et al. 2003, Leclerque and Kleespies 2008).

Both systems can translocate not only soluble effectors but also effectors containing transmembrane domains (TMDs), hereafter referred to as transmembrane effectors (TMEs). Membrane integration enables the spatial confinement of effector function, allowing TMEs to exert their activity at specific subcellular locations where critical host processes are regulated, thereby enhancing functional specificity (Hicks and Galán 2013, Escoll et al. 2016). The molecular mechanisms underlying the secretion of TMEs via the T4SS, as well as their subsequent targeting and integration into host membranes remain poorly understood. Special to TMEs is the existence of two independent secretion signals. The C-terminal T4SS-secretion signal facilitates translocation via the T4SS while the hydrophobicity of TMDs signals targeting to the bacterial inner membrane (IM). It was recently shown that more hydrophobic TMEs are secreted via a two-step secretion pathway with an IM intermediate while TMEs of intermediate hydrophobicity are directly secreted through the T4SS instead (Krampen et al. 2018, Malmsheimer et al. 2024). Also the pathway of targeting of TMEs to and insertion into host cell membranes may depend on their hydrophobicity so that different host mechanisms may be exploited by these specialized effectors.

Here, we aim to offer a comprehensive view of TME translocation by integrating TME hydrophobicity and topology predictions with the established requirements for type IV-dependent secretion and host membrane insertion pathways. Besides providing valuable insight into the preferred host membrane targets of T4SS-secreted TMEs, this review aims to establish a conceptual framework for the development of models that explain how bacterial TMEs can be accurately targeted and integrated into host cell membranes.

### Hijacking host membranes: the role of TMEs in pathogenesis

Several intracellular bacterial pathogens inject effector proteins into the host cell cytoplasm to promote bacterial survival. Type IV (T4SS) and Type III (T3SS) secretion systems are particularly well-characterized for mediating the delivery of these effectors. Efficient modulation of host cell processes often requires a spatially restricted activity of effector proteins at specific subcellular compartments. A number of bacterial effectors have evolved mechanisms to associate with host membranes, thereby enhancing their local concentration and functional specificity.

Membrane targeting can be achieved through diverse strategies. Some effectors undergo host-mediated posttranslational lipid modifications, which promote membrane attachment, exemplified by *L. pneumophila* LegG1 (Price et al. 2010, Ivanov et al. 2010) and *Salmonella enterica* SifA (Hicks and Galán 2013). Other effectors associate with the membrane through direct interactions with either integral membrane proteins or specific host membrane lipids. *Legionella* secretes a broad repertoire of phosphatidylinositol phosphate (PIP)-binding effectors, including SidC, SidM, and SetA, many of which harbor PI3P- or PI4P-specific lipid-binding domains that enable selective targeting to host membranes (Swart and Hilbi 2020, Lockwood et al. 2022). Similarly, the *Salmonella* effector SteA has been shown to bind PI4P, thereby anchoring itself to host cell membranes (Domingues et al. 2016).

Beyond peripheral targeting, some effector proteins stably insert into membranes via intrinsic hydrophobic TMDs (Krampen et al. 2018; Table 1). Bacterial TMEs function at membrane interfaces and mediate a range of biological processes inaccessible to cytoplasmic effectors (Godlee and Holden 2023). A key function of TMEs is the modulation of vesicular trafficking along secretory and endocytic pathways, critical for the formation and maintenance of the PCV. Many TMEs integrate into the membrane of the PCV and recruit Rab GTPases to promote vesicle tethering and fusion, supporting nutrient acquisition (Escoll et al. 2016). In *Legionella*, TMEs such as LidA and PieE have been shown to interact with multiple Rab GTPases, facilitating the recruitment and fusion of endoplasmic reticulum (ER)-derived vesicles with the *Legionella*-containing vacuole (LCV) (Meng et al. 2013, Mousnier et al. 2014). Similarly, *Chlamydia* TMEs, including Cpn0585 and CopS, bind host Rab proteins to modulate trafficking to the inclusion membrane (Rzomp et al. 2006, Cortes et al. 2007).

**Table 1:** T4ASS- and T4BSS-secreted TMEs and their target membrane in the host cell.

Name	Protein ID	Organism	Membrane localization after		Number of TMS	Function	Interacting proteins	References
			Ectopic expression	Infection				
MavN/DimB IroT	Lpg2815 Lpg2867	All *Legionella* species	–	LCV	8	Ferrous iron transporter for iron-acquisition by bacteria in the LCV; core effector also in *Rickettsiella*		Isaac et al. (2015), Christenson et al. (2019), Abeyrathna et al. (2019)
Ceg4	Lpg0096	*L. pneumophila*	ER	–	2	HAD protein-tyrosine phosphatase attenuates MAPK p38 signaling		Quaile et al. (2018)
LegU1	Lpg0171	*L. pneumophila*	ER	–	2	E3 ubiquitin ligase activity by forming Skp1-Cullin-F-box protein (SCF) complex; mediates ubiquitinational degradation of host chaperones BiP or BAT3	Skp1, BAT3, BiP	Ensminger and Isberg (2010), Zhao et al. (2025)
Ceg9	Lpg0246	*L. pneumophila*	ER	–	2	Regulating ER tubule and LCV formation	Rtn4 and Atlastin-1	Haenssler et al. (2015)
LidA	Lpg0940	*L. pneumophila*	ER ERGIC Golgi	–	1	Tethering of ER-derived vesicles to the LCV and promotion of Rab1 recruitment	Rab1, Rab6a, and Rab8	Derré and Isberg (2005), Meng et al. (2013)
Ceg23/LotB	Lpg1621	*L. pneumophila*	ER secretory pathway	LCV	1	OTU-type DUB deubiquinates Sec22b to facilitate noncanonical SNARE pairing	COPI and Sec22b	Kitao et al. (2020), Shin et al. (2020), Ma et al. (2020), (2023)
LegC3/PpeA	Lpg1701	*L. pneumophila*	ER (mammalian cells) Vacuole and PM (yeast)	–	2	Q-SNARE mimic; modulating endosomal trafficking; inhibitor of homotypic yeast vacuole fusion	VAMP4 LupA (metaeffector of LegC3)	Bennett et al. (2013), Shin et al. (2020), De Felipe et al. (2008)
LegC2/YlfB	Lpg1884	*L. pneumophila*	ER (mammalian cells) Vacuole (yeast)	–	1	Q-SNARE mimic; modulating endosomal trafficking; similar to *Chlamydia* IncA	VAMP4	Bennett et al. (2013), Shin et al. (2020), De Felipe et al. (2008)
LegC7/YlfA	Lpg2298	*L. pneumophila*	ER (mammalian cells) Vacuole (yeast)	–	1	Q-SNARE mimic; modulating endosomal trafficking; inducing ER–endosome fusion events; similar to *Chlamydia* IncA	VAMP4; ER-to-Golgi glycoprotein cargo adapter complex Emp47/46	Bennett et al. (2013), Shin et al. (2020), De Felipe et al. (2008), Glueck et al. (2021), O’Brien et al. (2015)
Lpg1751	Lpg1751	*L. pneumophila*	Vesicles of endomembrane trafficking (mammalian cells) Vacuole (yeast)	–	1	Promoting membrane fusion of lysosomes with LCV		Weigele et al. (2017)
PieE/LemB	Lpg1969	*L. pneumophila*	ER (mammalian cells) Golgi (yeast)	LCV	2	Modulation of vesicle trafficking, tethering of membranes and facilitating ER recruitment	Rab GTPases 1, 2a, 5c, 6a, 7, 10	Weigele et al. (2017), Mousnier et al. (2014)
MavE	Lpg2344	*L. pneumophila*	Vesicles	LCV	1	ER-mediated remodeling of the LCV and phagolysosomal evasion		Vaughn et al. (2021)
			LCV vesicles					
LepB	Lpg2490	*L. pneumophila*	ER	LCV	2	PI4 kinase generating PI(3,4)P to contribute PIP conversion on LCV; Rab1 GAP domain to deactivate Rab1; similar to SidF	Rab1	Dong et al. (2016), Ingmundson et al. (2007), Chen et al. (2007)
SidF	Lpg2584	*L. pneumophila*	ER	LCV	2	PI3P phosphatase modulating PIP levels on the LCV; neutralizing BNIP3 and Bcl-rambo to inhibit mitochondria-associated proapoptotic signaling	BNIP3 and Bcl-rambo	Dong et al. (2016), Hsu et al. (2012), Speir et al. (2017)
LecE	Lpg2552	*L. pneumophila*	ER cis-Golgi	LCV	5	Induces toxicity in yeast by activating phosphatidic acid (PA) phosphatase Pah1; influences diacylglycerol levels		Viner et al. (2012)
LncP	Lpw_31961	*L. pneumophila*	Mitochondrial inner membrane	Mitochondrial inner membrane	6	*L*. nucleotide carrier (Lnc) protein transporting ATP across mitochondrial membranes		Dolezal et al. (2012)
CBU0372	CBU0372	*C. burnetii*	ER	–	2	Fic family protein		Weber et al. (2013)
ElpA	CBUD1884	*C. burnetii* (Dugway)	ER	–	2	ER-localizing protein A; disrupting host cell secretory transport		Graham et al. (2015)
CBU0635	CBU0635	*C. burnetii*	Golgi vesicles of secretory pathway	–	3	Interferes with host secretory pathway		Carey et al. (2011)
CvpC	CBU1556	*C. burnetii*	CCV vesicular components		3	Coxiella vacuolar protein C		Larson et al. (2015)
CvpD	CBU1818	*C. burnetii*	CCV vesicular components		1	Coxiella vacuolar protein D		Larson et al. (2015)
CvpE	CBU1863	*C. burnetii*	CCV vesicular components		3	Coxiella vacuolar protein E		Larson et al. (2015)
CirC/OmpI/MceB	CBU0937	*C. burnetii*	Mitochondrial tubules	–	1	Porin at the outer bacterial membrane (Sec/SPI substrate SP) for the acquisition of an important metabolites in the CCV lumen; necessary for intracellular replication; T4bSS translocation contentious		Yang et al. (2025), Fielden et al. (2021), Crabill et al. (2018), Weber et al. (2013)
			Mitochondrial tubules					
MceC	CBU1425	*C. burnetii*	Mitochondrial tubules	–	1	Interacting with mitochondrial proteins of quality control machinery proteins	YME1L	Fielden et al. (2021)
			Mitochondrial tubules					
VceC	BAB1_1058	*Brucella abortus* str. 2308	ER		1	Induces ER stress by activating UPR resulting in proinflammatory host cell response	BiP/GRP70	Tsai et al. (2019), de Jong et al. (2008, 2013)
BspA	BAB1_0678	*Brucella abortus* str. 2308	ER		2–4	Interferes with host secretory pathway		Myeni et al. (2013), Tsai et al. (2019)
BspB	BAB1_0712	*Brucella abortus* str. 2308	ER ERGIC Golgi		2	Interferes with host secretory pathway; targets the conserved oligomeric Golgi (COG) tethering complex to redirect vesicular trafficking from Golgi to rBCV	COG tethering complex	Myeni et al. (2013), Miller et al. (2017)
BspD	BAB1_1611	*Brucella abortus* str. 2308	ER		1			Myeni et al. (2013), Tsai et al. (2019)
BspK	BAB2_0541	*Brucella abortus* str. 2308	ER		3			Myeni et al. (2013), Tsai et al. (2019)
BspC	BAB1_0847	*Brucella abortus* str. 2308	Golgi		1			Myeni et al. (2013)
BspF	BAB1_1948	*Brucella abortus* str. 2308	Plasma membrane protrusions Cytosol		1			Myeni et al. (2013)

In addition to Rab GTPases, the regulation of SNAREs and PIP lipids is critical for PCV remodeling and for preventing fusion with degradative lysosomes. Several *Legionella* TMEs, including LegC2, LegC3, and LegC7 (Bennett et al. 2013), as well as the *Chlamydia* effector IncA (Ronzone and Paumet 2013), harbor coiled-coil domains with high homology to SNARE proteins. These domains enable the TMEs to either facilitate SNARE-mediated membrane fusion via homo- or heterotypic interactions, or interfere with endogenous SNARE complexes to inhibit vacuolar fusion with lysosomes. PIPs also play a crucial role in defining membrane identity and directing vesicular trafficking by recruiting Rab GTPases. In *Legionella*, remodeling the PIP composition of the LCV membrane is essential for evading lysosomal fusion. To achieve this, the bacterium secretes the TMEs SidF and LepB, which function cooperatively to enrich the LCV membrane with PI4P, a lipid enriched in the Golgi apparatus and secretory vesicles (Hsu et al. 2012, Dong et al. 2016, Swart and Hilbi 2020, Vormittag et al. 2023).

TMEs also play key roles in establishing and maintaining membrane contact sites between distinct organelles, serving as tethers that facilitate nonvesicular exchange of lipids and ions (Helle et al. 2013). In *Chlamydia*, the TMEs IncD and IncV contribute to ER-inclusion membrane contact site formation by interacting with the ceramide transfer protein CERT and its ER-resident partners VAPA/B (Agaisse and Derré 2014, Murray et al. 2017). Similarly, several *Legionella* TMEs including PieE, Ceg9, and MavE have been implicated in tethering ER-derived tubules to the LCV by engaging with host adaptor proteins (Mousnier et al. 2014, Haenssler et al. 2015, Vaughn et al. 2021). Other pathogens also exploit membrane contact sites to localize to the vicinity of the Golgi apparatus. The *Salmonella* TMEs SseF and SseG form a trimolecular complex with ACBD3 and the Golgi-resident protein Giantin to anchor the *Salmonella*-containing vacuole to the Golgi region (Yu et al. 2016). Likewise, *Brucella* utilizes the T4ASS effector BspB to engage the conserved oligomeric Golgi tethering complex, promoting stable association of the *Brucell*a-containing vacuole with Golgi-associated membranes (Miller et al. 2017).

Another functional category of TMEs involves the transduction of signals or transport of molecules across host membranes, thereby modulating intercompartmental communication, ion homeostasis, and nutrient acquisition. One well-characterized example is the *Escherichia coli* TME Tir, which is inserted into the host plasma membrane to serve as a receptor for bacterial intimin. Binding of Tir to intimin triggers localized actin polymerization at the site of bacterial attachment to form typical lesions called actin pedestals (Frankel and Phillips 2008). In addition to signal transduction, some TMEs function as transporters. For instance, the *Legionella* TME LncP integrates into the inner mitochondrial membrane and acts as a nucleotide carrier, facilitating ATP efflux from the mitochondrial matrix (Dolezal et al. 2012). Another TME, MavN, which is a highly conserved core effector within the *Legionella* genus, integrates into the LCV membrane to function as a ferrous iron transporter. This activity is critical for intravacuolar iron acquisition and intracellular bacterial replication (Isaac et al. 2015, Christenson et al. 2019).

Altogether, membrane integration of TMEs, delivered by both T4SS and T3SS, is a key pathogenic strategy, enabling intracellular bacteria to subvert host membrane dynamics and establish a replicative niche.

### Biophysical features of T4-secreted membrane effector proteins

The biophysical properties of TMEs are likely to dictate the route for type IV-dependent translocation and insertion into the host membrane. Building on previous systematic *in silico* analyses characterizing such properties for T4BSS-secreted TMEs from *L. pneumophila* strain Philadelphia 1 and *C. burnetii* (Krampen et al. 2018, Malmsheimer et al. 2024), we extended this approach to a broader dataset of T4-secreted TMEs. Our analysis includes all known T4BSS effectors from multiple *L. pneumophila* strains (Philadelphia 1, 2300/99 Alcoy, Paris, Corby, and Lens), *L. longbeachae*, and *C. burnetii* (Bi et al. 2013), as well as T4ASS substrates from *Brucella abortus* (Sankarasubramanian et al. 2016). The *Brucella* effector dataset of Sankarasubramanian et al. (2016), generated by the S4TE prediction algorithm, excluded four experimentally validated and six putative T4ASS substrates (Myeni et al. 2013) due to its predefined scoring threshold; these effectors were additionally incorporated into our analysis. Moreover, T4ASS core effectors of *Rickettsia* spp. (Aspinwall and Brayton 2022), and *Bartonella henselae* (Bi et al. 2013) were also included, providing a more comprehensive assessment across both T4SS subtypes (Table S1).

This analysis evaluated both the structural membrane topology of TMEs, such as transmembrane segments (TMS) count and extramembranous domain length, and the biophysical potential of TMEs for membrane insertion or binding to the signal recognition particle (SRP), based on the sequence hydrophobicity. As discussed in detail later, SRP is a key determinant of membrane targeting in both eukaryotes and prokaryotes.

**Figure 1:**
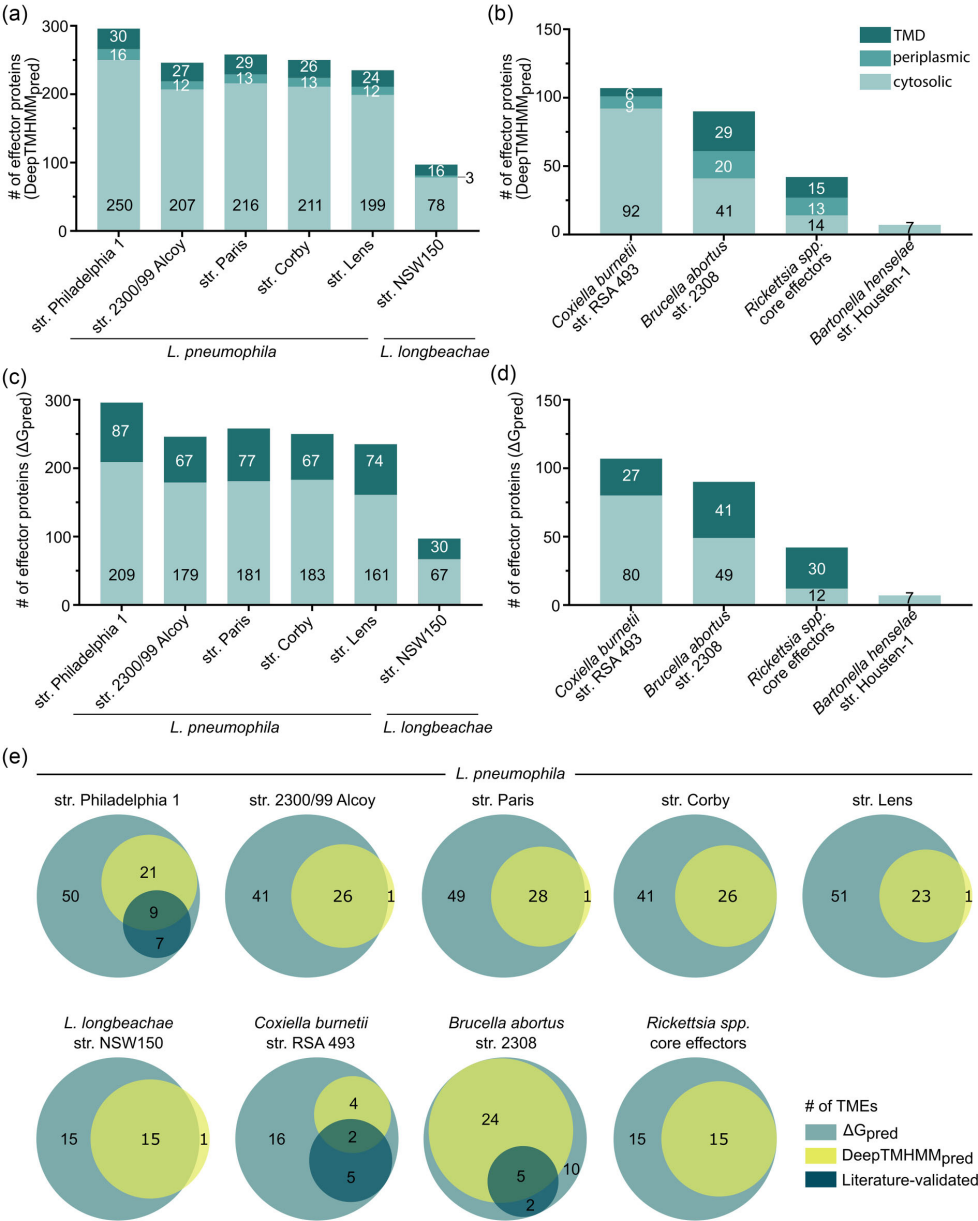
*In silico* prediction and characterization of T4ASS**-** and T4BSS**-**secreted TMEs. (a–d) Classification of predicted effectors as transmembrane, periplasmic, or cytosolic based on TMD or SP predictions. TMEs were predicted using two complementary approaches: (a and b) DeepTMHMM for transmembrane topology analysis and (c and d) the ΔG predictor to assess membrane integration propensity, applying a sequence window size of 18–35 aa including length correction and a threshold of <1.5 kcal/mol to define *bona fide* TMS. (e) Venn diagram comparing TMEs identified by DeepTMHMM and the ΔG predictor with experimentally validated TMEs (Table 1). Analyses were performed on T4BSS**-**secreted effectors from *L. pneumophila* strains, *L. longbeachae*, and *C. burnetii*; and T4ASS-secreted effectors from *B. abortus, B. henselae*, and core *Rickettsia* spp. effectors (Table S1).

To predict and classify transmembrane α-helical effector proteins, the deep learning protein language model-based algorithm DeepTMHMM was employed (Hallgren et al. 2022). Across various *L. pneumophila* strains and *L. longbeachae*, ~10% of T4BSS-secreted effectors were predicted to contain TMDs, and ∼5% possessed cleavable signal peptides (SPs) for periplasmic translocation (Fig. 1a). In *C. burnetii*, the predicted proportion was lower, with 6% classified as TMEs and 8% as SP-containing effectors (Fig. 1b). By contrast, a larger fraction of T4ASS substrates from *B. abortus* and *Rickettsia* spp. were predicted to contain TMDs (30%) and SPs—22% in *B. abortus* and 31% in *Rickettsia* spp. (Fig. 1b).

To complement topology-based predictions, the thermodynamic favorability of membrane insertion of T4-secreted effector proteins was assessed using the ΔG prediction tool (Hessa et al. 2007). α-Helical membrane proteins are evolutionarily optimized to match the physicochemical properties of lipid bilayers, with TMSs characterized by specific amino acid (aa) composition, length, and amphiphilicity. This optimization is reflected in the apparent Gibbs free energy of membrane insertion (ΔG_app_), which can be calculated for sequence windows of 10–40 aa based on empirical data (Hessa et al. 2007). Applying a ΔG_app_ threshold of <1.5 kcal/mol for putative TMSs (18–35 aa windows including length correction), ~30% of T4BSS effectors from the analysed *L. pneumophila* and *L. longbeachae* strains as well as *C. burnetii* were predicted to contain TMDs (Fig. 1c and d). In contrast, a higher proportion of T4ASS effectors from *B. abortus* and *Rickettsia* spp. classified as putative TMEs, with 45% and 70%, respectively (Fig. 1d).

**Figure 2:**
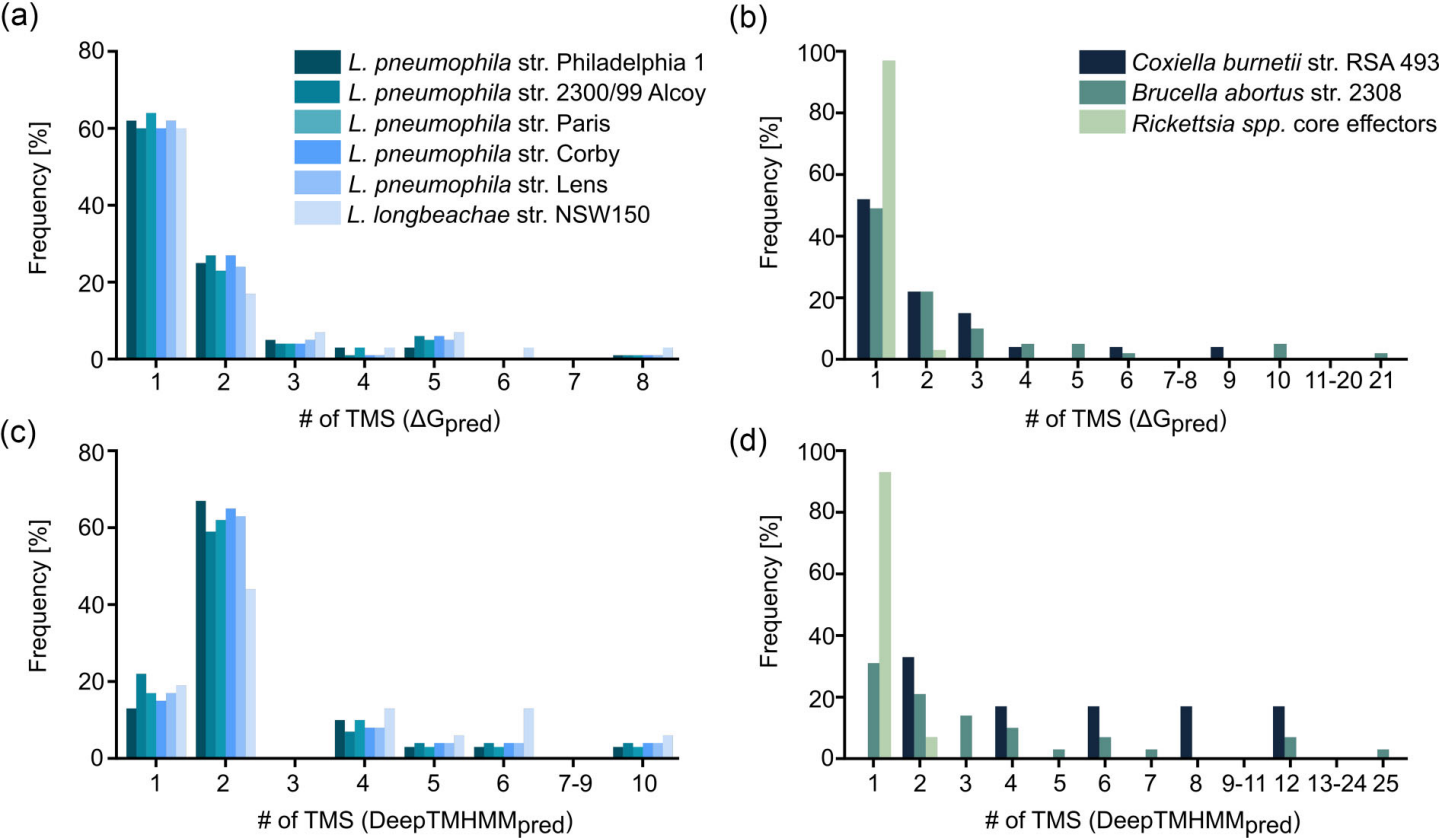
Predicted number of TMS among T4ASS**-** and T4BSS**-**secreted TMEs. TMS were predicted using (a and b) the ΔG predictor to evaluate membrane insertion propensity (window size 18–35 aa, <1.5 kcal/mol threshold) and (c and d) DeepTMHMM for transmembrane topology prediction. Analyses were performed on T4BSS**-**secreted effectors from *L. pneumophila* strains, *L. longbeachae*, and *C. burnetii*; and T4ASS**-**secreted effectors from *B. abortus, B. henselae*, and core *Rickettsia* spp. effectors (Table S1).

The above results indicate that the proportion of predicted TMEs varies depending on the computational tool employed, reflecting differences in methodological focus (Fig. 1e). The ΔG prediction tool assesses the thermodynamic favorability of membrane insertion based on the membrane integration propensities of individual aa residues within the protein, whereas DeepTMHMM emphasizes membrane protein topology, including the discrimination of SPs from *bona fide* TMSs. As a result, the ΔG predictor often misclassified the hydrophobic core of SPs as the first TMS, contributing to an overrepresentation of single-TMS predictions, particularly in *Legionella* (60%; Fig. 2a), *Coxiella* and *Brucella* (50% each; Fig. 2b). In contrast, DeepTMHMM failed to predict several experimentally validated TMEs, such as *Legionella* TMEs Ceg4, LidA, LegC7, MavE, Lpg1751, LepB, or SidF (Table 1; Fig. 1e), likely due to its training on a limited dataset of classical α-helical membrane proteins, which do not represent effector proteins delivered via bacterial secretion systems and lack considerations of membrane insertion energetics. Both prediction tools, in particular the ΔG predictor, accurately identified experimentally validated TMEs from *L. pneumophila* str. Philadelphia 1, *C. burnetii*, and *B. abortus* (Table 1; Fig. 1e). Within the limits of literature-based validation, no false-positive predictions were detected, although only 17% (*Legionella* and *Brucella*) to 26% (*Coxiella*) of the predicted TMEs have been experimentally examined for their subcellular localization in host cells.

Comparative analysis of the predicted T4A- and T4B-secreted TMEs revealed a general predominance of low-complexity transmembrane topologies. Approximately 80% of these effectors possess only one or two TMS, with the exact distribution influenced by the prediction method used (Fig. 2a–d). Similar findings have been reported for T3SS substrates (Krampen et al. 2018), suggesting that both T4SS and T3SS preferentially translocate membrane effectors with simple topologies. A small subset of T4BSS effectors exhibited more complex architectures, including proteins with up to 12 predicted TMS. Remarkably, a *B. abortus* effector was predicted to contain as many as 21–25 TMS, depending on the prediction method. In contrast, nearly all predicted TMEs from *Rickettsia* spp. were single-span membrane proteins, with only one exception harboring two TMS (Fig. 2a–d).

**Figure 3:**
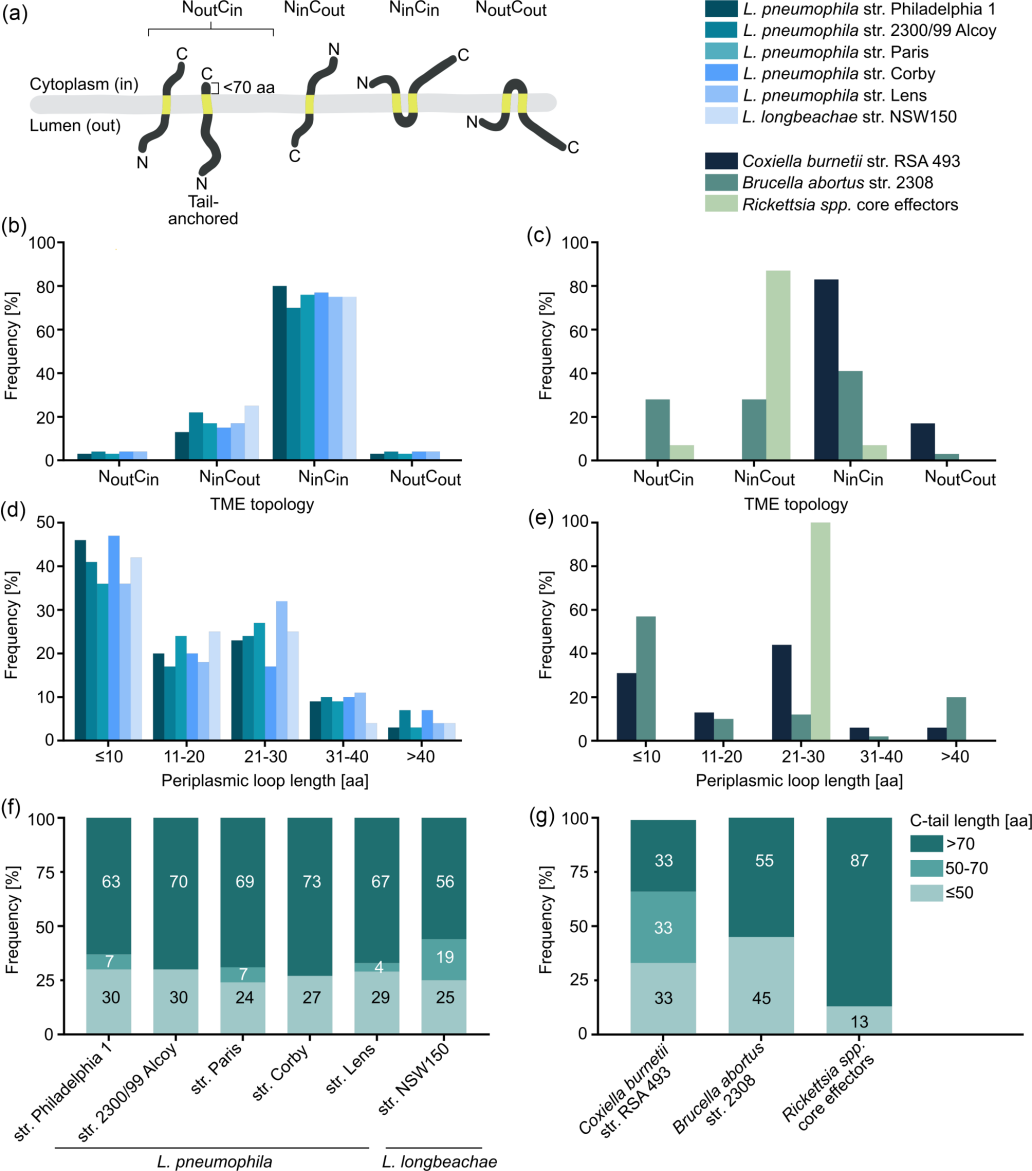
Topology characteristics of T4ASS**-** and T4BSS**-**secreted TMEs predicted by DeepTMHMM. (a) Schematic representation of membrane protein topologies. The orientation can vary depending on the number (*n*) of predicted TMS: *n = 2x + 1* for N_out_C_in_ or N_in_C_out_ topologies, and *n = 2x* for N_in_C_in_ or N_out_C_out_ topologies. (b–g) Frequency analysis of (b and c) predicted membrane topologies, (d and e) periplasmic loop lengths, and (f and g) C-terminal tail lengths of T4BSS**-**secreted TMEs from (b, d, and f) *L. pneumophila* strains, *L. longbeachae*, and (c, e, and g) *C. burnetii*; and T4ASS**-**secreted TMEs from *B. abortus, B. henselae*, and core effectors of *Rickettsia* spp. (Table S1).

Moreover, DeepTMHMM analysis revealed that the majority of T4BSS-secreted TMEs from *Legionella* spp. and *C. burnetii* (70%–80%) exhibit an N_in_C_in_ membrane topology (Fig. 3a–c). This means that the amino (N)- and carboxy (C)-termini of the TMEs are both localized intracellularly, that is, either in the bacterial or the host cytoplasm. This topology appears to be a distinguishing feature of T4BSS-dependent TME secretion, as it is prevalent in *Legionella* and *Coxiella*. In contrast, T4ASS-secreted TMEs exhibit greater topological variability, as shown for *B. abortus* effectors (Fig. 3c). Furthermore, the single-pass TMEs from *Rickettsia* spp. predominantly adopt an N_in_C_out_ topology (87%), that is, with an extracellular C-terminus, either in the bacterial periplasm or the PCV lumen (Fig. 3a and c).

DeepTMHMM also predicted that most multipass TMEs possess very short periplasmic loops, with ~80% measuring <30 aa and half of these shorter than 11 aa (Fig. 3d and e). This structural feature appears to be conserved across both T4A- and T4B-secreted TMEs, highlighting the functional advantage of low-complexity membrane topologies. Supporting this, over 60% of T4A- and T4B-secreted TMEs contain extended C-terminal tails longer than 70 aa (Fig. 3f and g), yet only a minority are predicted to face the periplasm. This suggests that most TMEs avoid the need for complex translocation of large TMS-flanking domains across the bacterial IM. Such simplicity may not only support efficient secretion but also facilitate rapid membrane integration in host cells (Smalinskaitė and Hegde 2023).

**Figure 4:**
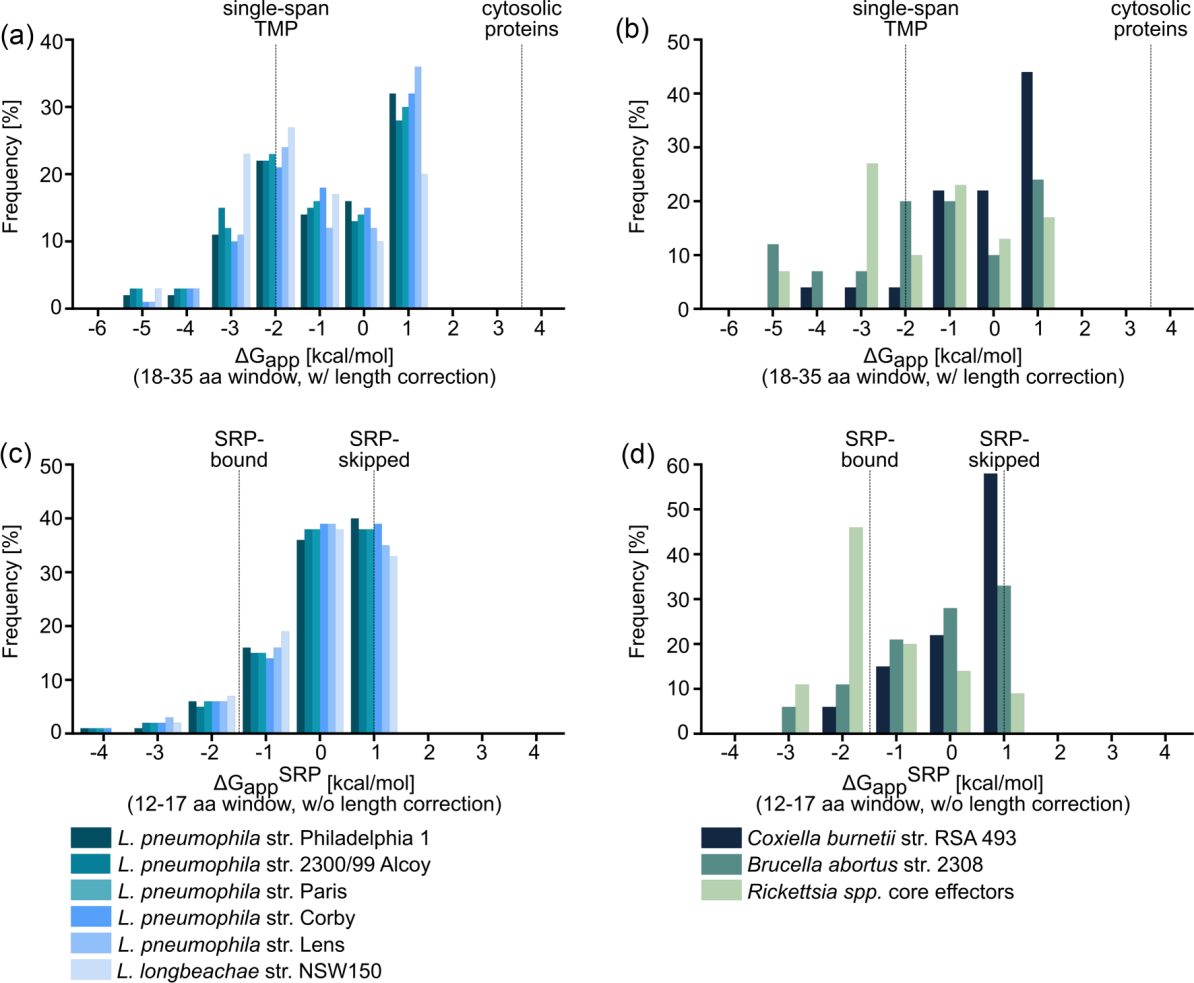
Membrane integration propensity and SRP**-**targeting potential of T4ASS**-** and T4BSS**-**secreted TMEs. T4BSS**-**secreted effectors from (a and c) *L. pneumophila* strains, *L. longbeachae*, and (b and d) *C. burnetii*, and T4ASS**-**secreted effectors from *B. abortus, B. henselae*, and core *Rickettsia* spp. effectors were analysed using the ΔG predictor (Table S1). (a and b) Distribution of the calculated membrane integration propensity (ΔG_app_) of TMS in predicted TMEs, compared to reference values for classical single**-**span transmembrane proteins and cytosolic proteins (Hessa et al. 2007). For each TME, only the segment with the lowest ΔG_app_ (calculated over a 18–35**-**aa window with length correction) is shown. (c and d) Analysis of SRP**-**dependent membrane targeting potential (ΔG_app_^SRP^) of predicted TMEs, compared to known SRP substrates and substrates, where the first TMS was skipped by SRP (Schibich et al. 2016). For each TME, the most hydrophobic SRP**-**targeting segment was determined using a 12–17**-**aa window without length correction.

All T4SS-secreted TMEs identified by the ΔG prediction tool were further analysed for their membrane integration propensity based on their minimal ΔG_app_ values for a 18–35-aa sequence window including corrections for length, and compared to reference ΔG_app_ distributions for single-spanning membrane proteins and soluble proteins (Hessa et al. 2007; Fig. 4a and b). T4B-secreted TMEs from *Legionella* spp. roughly fall into two groups regarding their ∆G_app_ values: one group with moderate ΔG_app_ values larger than 0 kcal/mol, and a second, more hydrophobic group with ΔG_app_ below 0 kcal/mol, aligning with values typical for single-pass membrane proteins, which peak at –2 kcal/mol (Fig. 4a). A similar distribution was observed for T4A-secreted TMEs of *B. abortus* and *Rickettsia* spp., though slightly shifted towards lower ∆G_app_ values (Fig. 4b). In contrast, *C. burnetii* T4B-secreted TMEs formed a single group with intermediate ∆G values (ΔG_app_ between –1 and +1 kcal/mol), consistent with previous findings for T3SS substrates that lie between single-span TM and soluble proteins (Krampen et al. 2018).

To assess the potential for SRP-dependent targeting of T4A- and T4B-secreted TMEs to the bacterial IM, ΔG_app_^SRP^ was calculated using a sequence window of 12–17 aa without length correction (Fig. 4c and d). These parameters reflect SRP recognition motifs, which typically consist of short, hydrophobic segments, with a ΔG_app_ between −2 and −1 kcal/mol (Schibich et al. 2016, Krampen et al. 2018). In contrast, N-terminal TMS skipped by SRP of otherwise SRP-targeted proteins exhibit higher ΔG_app_ values, peaking at +1 kcal/mol (Schibich et al. 2016). Most T4BSS TMEs from *Legionella* and *C. burnetii*, as well as T4ASS TMEs from *B. abortus* displayed their lowest ΔG_app_^SRP^ values between 0 and +1 kcal/mol, suggesting that ∼80 % escape SRP recognition, which is consistent with prior findings for T3SS substrates (Krampen et al. 2018). In contrast, ∼80 % of *Rickettsia* T4ASS TMEs and ∼20 % of *Legionella* and *C. burnetii* T4BSS TMEs displayed ΔG_app_^SRP^ values that are within the SRP–recognition range (Fig 4c and d). These findings indicate that T4-secreted TMEs can be classified into two distinct groups based on their hydrophobicity and SRP-recognition potential. The first comprises highly hydrophobic TMEs resembling classical single-span membrane proteins that are likely recognized by the SRP and targeted to the bacterial IM prior to type IV-dependent secretion (Malmsheimer et al. 2024). The second includes moderately hydrophobic TMEs that avoid classical membrane targeting pathways inside the bacterium, enabling their direct translocation via the T4SS (Krampen et al. 2018).

Altogether, the *in silico* analyses of T4ASS- and T4BSS-secreted effector repertoires revealed significant variability in the number of predicted TMEs across different computational tools, with the percentage of effector proteins destined for insertion into host cell membranes ranging from ~10% (DeepTMHMM) to 30% (ΔG predictor). Among these, efficient recognition and translocation by the T4SS may be favored by moderate TMS hydrophobicity and a relatively simple membrane topology, typically comprising one or two TMSs, short extramembranous loops, and a predominant N_in_C_in_ orientation. Nevertheless, the secretion of more complex TMEs, characterized by multiple TMSs or higher overall hydrophobicity, also appears to be feasible.

### The effector translocating T4SS

**Figure 5:**
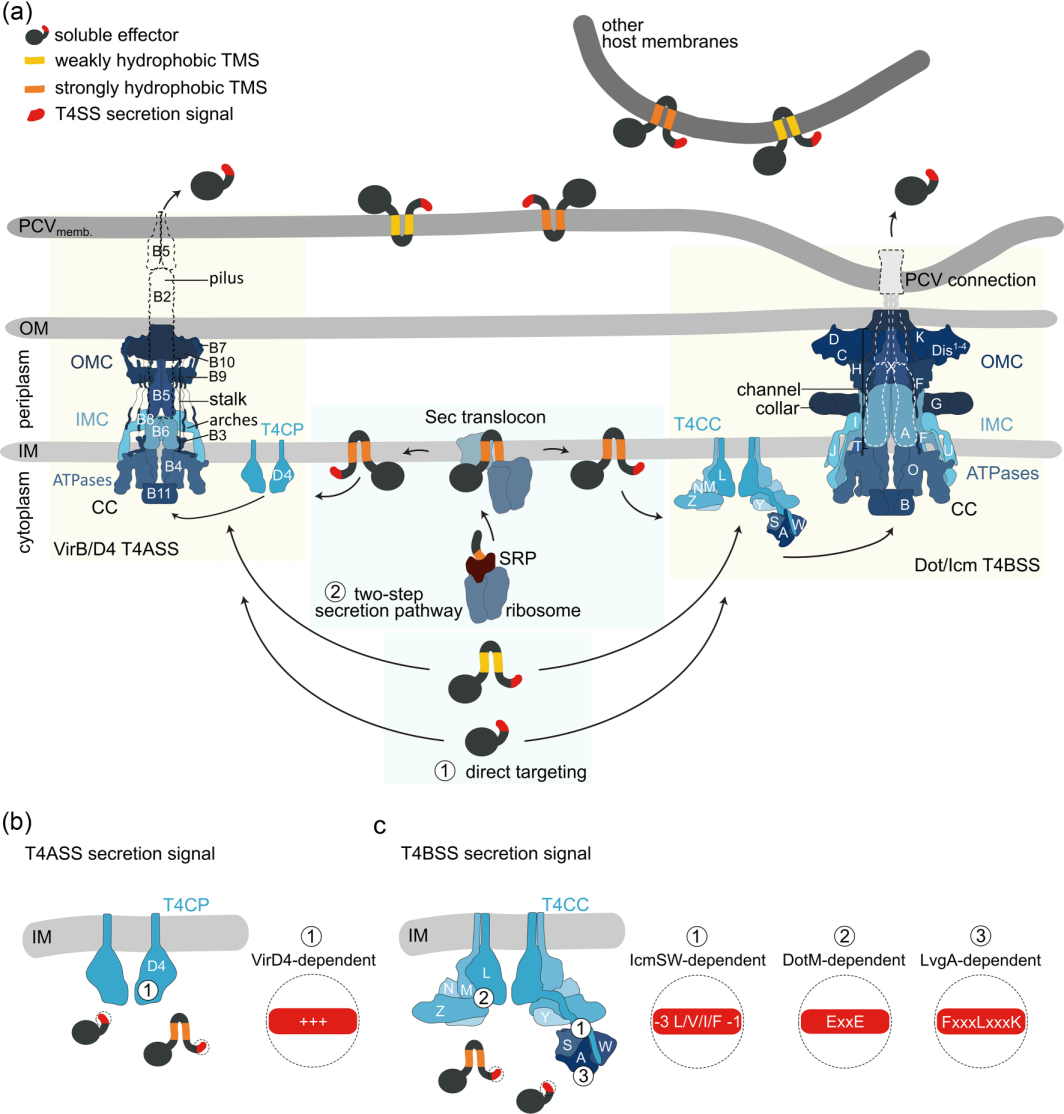
Targeting and translocation mechanism of soluble and membrane effectors of the VirB/D4 T4ASS and the Dot/Icm T4BSS. (a) Model of TMS**-**dependent targeting discrimination of TMEs of the T4SS. (1) TMEs with a weak hydrophobic TMS and soluble effectors are directly targeted to the T4CP/T4CC and are further translocated by the CC. (2) TMEs with a strong hydrophobic TMS are targeted to and inserted into the bacterial IM via the SRP and Sec translocon, followed by recognition of the IM intermediate by the T4CP/T4CC, subsequent extraction from the IM and translocation by the T4SS into the host cell. Shown on the left is the structural model of the protein components of the VirB/D4 type IV A secretion system (T4ASS) based on the structure of the plasmid R388 system (Macé et al. 2022a). The CC consists of the OMC proteins VirB7, VirB9, and VirB10 and the IMC proteins VirB3, VirB6–VirB5 (stalk), VirB8 (arches), and the two ATPases VirB11 and VirB4. The pilus is made up of the VirB2 subunits and the tip protein VirB5. One additional component is the T4CP VirD4. On the right is the structural model of the protein components of the Dot/Icm type IV B secretion system (T4BSS) based on the structure of the *L. pneumophila* system (Ghosal et al. 2017, Kwak et al. 2017, Chetrit et al. 2018, Meir et al. 2018, Dutka et al. 2026, Yue et al. 2025). The CC consists of the OMC proteins DotC, DotD, DotK, Dis1–4, the PR proteins DotF and DotH, the collar DotG, the channel proteins IcmX, DotA, and/or IcmF and the IMC proteins DotI, DotJ, DotU, and IcmT, and the two ATPases DotB and DotO. The T4CC is composed of the T4CP DotL, the structural components DotM, DotN, DotY, and DotZ, and the chaperon**-**like adaptor proteins IcmS, IcmW, and LvgA. Additional proteins that are also part of the T4BSS are not shown here. The dotted lines indicate the structure of the actively secreting systems and the closed lines the inactive systems. (b) T4ASS effector targeting group based on the C**-**terminal secretion signal. (1) The positively charged C**-**terminal secretion signal of T4ASS effector proteins is recognized by VirD4. (c) T4BSS effector targeting groups based on the C**-**terminal secretion signal. (1) IcmSW**-**dependent effector proteins have a hydrophobic residue in the last three C**-**terminal residues (_−3_ L/V/I/F_−1_) or harbor an internal secretion signal, which is still undefined and not shown here. (2) DotM**-**dependent effector proteins carry a Glu**-**rich secretion signal, the so**-**called E-block motif (ExxE). (3) LvgA-dependent effector proteins harbor the secretion signal FxxxLxxxK at the C-terminus. TMS. TMD. TME. Type IV coupling protein (T4CP). Type IV coupling complex (T4CC). Core complex (CC). PCV. Outer membrane (OM). Outer membrane complex (OMC). Periplasmic ring (PR). Inner membrane (IM). Inner membrane complex (IMC).

Understanding the mechanism by which TMEs are secreted through the T4SS requires knowledge of the structural organization of the translocation machinery. The classification of the T4SS into T4ASS and T4BSS is based on homology of the structural components (Christie and Vogel 2000). T4ASSs are homologous to the Ti plasmid-encoded VirB/D4 (virulence) secretion system of *Agrobacterium tumefaciens*, which allows conjugation and effector translocation into plant cells. The effector translocating Dot/Icm T4BSS group is homologous to the IncI plasmid-encoded conjugational Tra system (Komano et al. 2000, Wilkins and Thomas 2000, Lawley et al. 2003). This review focuses on the effector translocating systems that are involved in maintaining intracellular survival of pathogens in host cells. To highlight similarities and differences, we will compare the structures of the T4ASS R388 and pKM101, and the T4BSS Dot/Icm of *L. pneumophila*, since these are the best studied complexes (Fig. 5a). For simplicity, only the Vir and Dot/Icm nomenclature will be used.

Both T4SSs span across the inner (IM) and outer (OM) bacterial membrane and connect to the host cell membrane (Low et al. 2014, Vincent et al. 2006, Chetrit et al. 2018, Böck et al. 2021; Fig. 5a). Both consist of two major parts: the core complex (CC), forming the translocation channel, and the type IV coupling protein (T4CP), which serves as an effector recruitment platform. In the T4BSS, the T4CP interacts with additional proteins forming the type IV coupling complex (T4CC) (Christie et al. 2005, Vincent et al. 2006, Fronzes et al. 2009a, Low et al. 2014, Kitao et al. 2022, Zehra et al. 2023), highlighting the higher complexity of the Dot/Icm T4BSS system compared to the T4ASS. This is also reflected in the size of the translocation machinery (400 Å for T4BSS vs. 200 Å for T4ASS) (Low et al. 2014, Macé et al. 2022a, Zehra et al. 2023), as well as in the number of components (30 Dot/Icm proteins in T4BSS vs. 12 VirB/D4 proteins in T4ASS).

#### The T4CP/complex

The T4CP VirD4/DotL is the primary contact site for substrate recruitment to the T4SS (Kwak et al. 2017, Álvarez-Rodríguez et al. 2020, Kitao et al. 2022, Costa et al. 2024; Fig. 5b and c). The N-terminal TMD of this hexameric ATPase participates in the formation of an IM channel while the nucleotide-binding domain provides energy for system assembly and effector targeting to the CC (Kerr and Christie 2010, Vincent et al. 2012, Meir et al. 2020, Costa et al. 2021). The all-alpha domain is relevant for substrate binding and the long unfolded C-terminal tail of DotL, which is not present in VirD4, was shown to bind the heterodimer IcmSW and interacts with LvgA (Sutherland et al. 2012, Xu et al. 2017). IcmS, IcmW, and LvgA are small acidic cytoplasmic proteins that bind effector proteins (Bardill et al. 2005, Ninio et al. 2005, Vincent and Vogel 2006, Cambronne and Roy 2007). The flexible interaction with the DotL extension allows IcmSW and LvgA to act as chaperone-like adaptor proteins (Segal and Shuman 1997, Zuckman et al. 1999, Coers et al. 2000). IcmSW swings at the base of the T4CC allowing the bound effector to be positioned into the DotL channel or be handed over to the CC (Meir et al. 2020). The effector recruitment platform T4CC of the Dot/Icm T4BSS not only consists of the T4CP DotL and the adaptor proteins, but also includes the proteins DotM, DotN, DotY, and DotZ (Kwak et al. 2017, Meir et al. 2018, 2020, Macé et al. 2022a). DotM interacts with DotL via the IM-anchored TMD (Vincent et al. 2012). Together with the cytoplasmic proteins DotN, DotY and DotZ, DotM forms a cavity where the Glu-rich C-terminal end of effector proteins can insert and interact with DotM (Meir et al. 2018, 2020, Macé et al. 2022a). The T4CP relation to the CC is still unclear, since the coupling protein/complex was never visualized together with the main translocation channel. This suggests that the interaction is transient and only exists during active secretion upon host membrane connection (Chetrit et al. 2018, Li et al. 2019).

#### The core complex

The CC, which appears as a ring with a central pore, represents the ATP-powered translocation channel for effector export into the host cell (Kubori et al. 2014). The inner membrane complex (IMC) is anchored to the IM and extends into the cytoplasm and the periplasm (Fig. 5a). The ATPases VirB4/DotO and VirB11/DotB associate to the IMC on the cytoplasmic side (Vincent et al. 2006, Ghosal et al. 2017, Chetrit et al. 2018, Park et al. 2020, Khara et al. 2021, Macé et al. 2022a), providing the energy for system assembly and substrate translocation (Vogel et al. 1998, Sagulenko et al. 2001, Atmakuri et al. 2004, Sexton et al. 2004, 2005). VirB4/DotO presents as a hexamer of dimers with a centric pore (Chetrit et al. 2018, Macé et al. 2022a). VirB11/DotB assembles into an hexameric structure and docks centrally onto the base of VirB4/DotO (Chetrit et al. 2018, Prevost and Waksman 2018). The ATPase complex creates the cytoplasmic translocation channel connecting to the channel spanning the IM. Membrane association of the cytoplasmic ATPase complex is mediated by the membrane receptors VirB3/IcmT and VirB8_tail_/DotIJ (Low et al. 2014, Macé et al. 2022a, Kuroda et al. 2015, Vijayrajratnam et al. 2024). In the VirB/D4 T4ASS system, the VirB8 protein extends from the IM into the periplasm and forms the arches around the central stalk density (Macé et al. 2022a). The base of the stalk is composed of the VirB6 pentamer, while the tip of the stalk consists of the pentameric VirB5 in the inactive system.

In the Dot/Icm T4BSS, two IM proteins may occupy the space of VirB6, forming the central hollow cylinder in the IM for effector translocation. One is IcmF, homologous to the channel-forming T6SS TssM (Durand et al. 2012, Dutka et al. 2026). DotU, the homolog of T6SS TssL, is supposed to be associated with the cylinder. The other potential cylinder protein is DotA, which shares some structural features with VirB6 (Yue et al. 2025). DotA interacts with the base of IcmX, which was thought to form the plug density in the outer membrane complex (OMC) chamber, blocking the consecutive translocation channel for effector export (Chetrit et al. 2018, Ghosal et al. 2019, Dutka et al. 2026). IcmX is a pentameric protein homologous to VirB5 and is known to be exposed to the host cell (Macé et al. 2022a, Gomez-Valero et al. 2019). Moreover, a shortened form of IcmX was found in the supernatant, indicating that IcmX gets exported by the Dot/Icm T4BSS (Matthews and Roy 2000). Since VirB5 is closely related to pore-forming proteins, IcmX could fall into this category as well, making it a suitable candidate for interaction with the host cell membrane to create a translocation pore. Also DotA was found to be secreted into the supernatant (Nagai and Roy 2001). On the other hand, DotA was shown to be essential for intracellular survival of *Legionella* (Berger et al. 1994, Roy et al. 1998). Recent structural analyses showed that DotA together with IcmX most likely forms a pentameric protochannel with a narrow lumen, restricting effector translocation in the inactive state (Yue et al. 2025). In actively secreting T4BSS, the conformation of the DotA–IcmX changes, creating an extended channel across both bacterial membranes with a widened lumen, allowing effector proteins to pass through.

The stalk, or cylinder, is connected to the OMC, which can be divided into two layers with a chamber in the middle (Fronzes et al. 2009a; Fig. 5a). The O-layer with a dome structure connects to the OM, while the I-layer, called periplasmic ring (PR) in the Dot/Icm T4BSS, is localized in the periplasm (Fronzes et al. 2009a, Macé et al. 2022a, Ghosal et al. 2017, Durie et al. 2020, Sheedlo et al. 2021). In the VirB/D4 T4ASS, VirB7, VirB9, and VirB10 are the major components of the CC forming the OMC (Christie et al. 2005, Fronzes et al. 2009b, Low et al. 2014, Macé et al. 2022a). The respective homologs in the Dot/Icm T4BSS are DotD, DotH, and DotG (Vincent et al. 2006, Ghosal et al. 2017, 2019, Durie et al. 2020, Sheedlo et al. 2021). VirB10/DotG spans from the OM, forming the dome structure, through the periplasm to the IM, connecting the channel/stalk to the OMC (Chandran et al. 2009, Kubori et al. 2014, Ghosal et al. 2019, Macé et al. 2022b, Macé and Waksman 2024, Yue et al. 2025). The structure suggests that VirB10/DotG encases the stalk (VirB6–VirB5), or the channel (DotA–IcmX), in a cage-like manner. Further, DotG was shown to constitute the collar density in the periplasm, which is not present in the VirB/D4 T4ASS (Dutka et al. 2026, Macé et al. 2022a, Khara et al. 2021, Hu et al. 2019). Additional components of the OMC in the Dot/Icm T4BSS are DotC, DotF, DotK, and Dis 1–4 (Chetrit et al. 2018, Ghosal et al. 2019, Durie et al. 2020, Sheedlo et al. 2021, Yue et al. 2025).

In the active state of the Dot/Icm T4BSS, the dome region of the OMC widens, allowing the DotA–IcmX channel to expand across the OM and possibly beyond. Indeed, an additional 15 nm extension from the OM was observed, suggesting membrane connection to the PCV, which would allow effector delivery directly into the host cell (Yue et al. 2025). However, which protein exactly facilitates the connection of the Dot/Icm T4BSS to the host membrane is still unknown (Böck et al. 2021, Dutka et al. 2026). This interaction is supported by the dented membrane of the *Legionella* containing vacuole (LCV), which protrudes toward the Dot/Icm T4BSS at the poles of *L. pneumophila*. This led to the suggestion that the translocation occurs through a very short pilus-like structure. Unlike the Dot/Icm T4BSS, the VirB/D4 T4ASS has a pilus that allows direct contact with the host cell membrane in an actively secreting system (Waksman 2019). The pilus subunits VirB2 are first integrated into the IM (Costa et al. 2016), and subsequently extracted, together with a phospholipid, by the action of the ATPases VirB4 and VirB11 (Sagulenko et al. 2001, Kerr and Christie 2010). The VirB2 subunits associate in a five-base helical shape to assemble the pilus (Costa et al. 2016), a process that likely takes place between the stalk proteins VirB6 and VirB5 (Waksman 2019, Macé et al. 2022a, Costa et al. 2024). During elongation of the pilus, VirB5 acts as the pilus tip protein and is pushed out of the T4ASS (Aly and Baron 2007, Fronzes et al. 2009a), which makes VirB5 the host contact protein. This is further supported by the hydrophobic tip of VirB5, which is suitable for interaction with the PCV membrane (Macé and Waksman 2024). Whether it also has a translocon/insertase-like function at the host membrane, remains to be determined. In summary, despite structural differences, T4ASS and T4BSS are overall similar, underlining their conserved function as translocation machines.

### Membrane effector secretion through the T4SS

#### Targeting of T4SS membrane effectors

To be translocated into the host cells during intracellular survival, effector proteins first need to be targeted to the secretion system. It is generally recognized that effector proteins harbor a secretion signal to be specifically recruited to the respective translocation machinery (Alvarez-Martinez and Christie 2009, Hui et al. 2021). Several approaches have shown that there is no universal targeting sequence that identifies as the secretion signal for T4SS-translocated effector proteins. Rather, a pattern of aa distribution with polar properties was identified at the C-terminal end of these effectors (Vergunst et al. 2005, Hohlfeld et al. 2006, Burstein et al. 2009, Huang et al. 2011, Lifshitz et al. 2013). Consistent with a role in type IV-dependent secretion, deletion of this region reduced effector translocation (Luo and Isberg 2004, Voth et al. 2009). For T4BSS-dependent effectors of *L. pneumophila*, it was shown that small polar aas are favored in the last 20 residues of effector proteins, and at the very C-terminus, hydrophobic residues are preferred (Nagai et al. 2005, Kubori et al. 2008, Burstein et al. 2009). The negatively charged residues glutamate (E) and aspartate (D) were found to be depleted from the very C-terminus but enriched at positions further upstream, forming the so-called E-block motif (Huang et al. 2011). Not all known T4BSS-dependent effector proteins harbor an E-block motif, which gives a first hint that effector proteins can be divided into different groups based on the T4SS targeting mechanism. The E-block motif appears to be the predominant secretion signal in *L. pneumophila* TMEs, as it is present in 65% of these effectors (Malmsheimer et al. 2024). The described pattern of the secretion signal was also found in *C. burnetii* and *R. grylli* effectors, which both encode a Dot/Icm T4BSS closely related to *L. pneumophila* (Lifshitz et al. 2013, Larson et al. 2019). Besides the C-terminal secretion signal, an additional internal signal was described for some effector proteins, further supporting the existence of different targeting mechanisms (Zhu et al. 2011, Jeong et al. 2015). The C-terminal secretion signal of T4ASS-secreted effector proteins is often positively charged (Vergunst et al. 2005, Hohlfeld et al. 2006, de Jong et al. 2008, Oka et al. 2022). For the effector proteins of *Bartonella* spp. a second secretion signal, the Bep-intracellular delivery domain, was observed in addition to the positively charged C-terminus (Schulein et al. 2005). This shows that also in the less complex T4ASS, different secretion signals may exist. The observed physical properties of the aa at the different positions of the C-terminal end are common to soluble effector proteins and TMEs (Burstein et al. 2009, Lifshitz et al. 2013), suggesting a similar mechanism of recruitment to the T4SS.

Effector recognition is facilitated by the T4CC in Dot/Icm T4BSS and by the T4CP VirD4 in VirB/D4 T4ASS (Vincent et al. 2012, Kwak et al. 2017, Meir et al. 2018, 2020, Álvarez-Rodríguez et al. 2020, Costa et al. 2024; Fig. 5b and c). Effectors are classified into different groups based on their secretion signal pattern and recognition by T4CC components, irrespective of whether they are soluble or transmembrane proteins (Kitao et al. 2022). IcmSW-dependent effectors were reported to not harbor an E-block motif but are bound by the adaptor proteins IcmS and IcmW (Bardill et al. 2005, Ninio et al. 2005, Cambronne and Roy 2007; Fig. 5c). A consistent secretion signal that is recognized by IcmSW is not yet known, but a specific internal region of the TME SidG and the soluble effector SidJ were identified to be relevant for IcmSW-dependent translocation (Cambronne and Roy 2007, Jeong et al. 2015). Effectors with an E-block motif seem to be IcmSW-independent and rely on DotM for efficient translocation (Fig. 5c). The positively charged patches of the cytoplasmic part of DotM have been described as the recruitment platform for the negatively charged, acidic Glu-rich motif of T4BSS effectors (Meir et al. 2018). However, some effectors show IcmSW-dependency even though they possess an E-block motif (Malmsheimer et al. 2024). Yet, another group of T4BSS-secreted effectors depend on the third adaptor protein LvgA for targeting, which was only identified in *Legionella*. Binding of these effector proteins to LvgA is mediated by the FxxxLxxxK motif (Kim et al. 2020; Fig. 5c). It is likely that the existence of different recruitment components in the Dot/Icm machinery reflects the great functional and structural variety among *L. pneumophila, C. burnetii*, and *R. grylli* effectors.

This general principle for effector targeting poses the question if and how posttranslational targeting of TMEs via the C-terminal secretion signal is delimited from cotranslational targeting to the inner bacterial membrane (Fig. 5a). Sufficiently hydrophobic TMSs of T4SS TMEs signal IM insertion via the SRP, which precedes targeting to the T4SS based on the C-terminal secretion signal (Schibich et al. 2016, Krampen et al. 2018, Malmsheimer et al. 2024). Depending on the hydrophobicity of the TMSs, secretion of TMEs must thus allow for alternative secretion pathways. TMEs with moderate TMS hydrophobicity may be directly targeted to the T4SS, because SRP most likely does not recognize them as a substrate (Krampen et al. 2018; Figs 5c and d and 5a). T4BSS TMEs of *L. pneumophila* with highly hydrophobic TMSs were proposed to translocate via a two-step secretion pathway instead (Malmsheimer et al. 2024). In the first step, a highly hydrophobic TMS is thought to first insert into the bacterial IM, followed by recognition of the IM intermediate by the T4CC, subsequent extraction from the IM, and translocation of the TME into the host cell by the CC (Fig. 5a). This pathway follows from the immediate recognition of sufficiently hydrophobic TMS by SRP (Schibich et al. 2016), which guides the nascent polypeptide chain to the SRP-receptor and eventually to the SecY-translocon, facilitating IM integration [for detailed information about the biogenesis of membrane proteins in prokaryotes refer to the reviews (Luirink et al. 2012, Mercier et al. 2022)]. This two-step secretion pathway may also be utilized by TMEs with a similar hydrophobicity profile from other T4BSS-harboring pathogens such as *C. burnetii*, as well as from T4ASS-harboring pathogens like *B. abortus* and *Rickettsia* species (Fig. 4d). Alternatively, similar to T3SS TMEs of *Salmonella*, whose mistargeting to the IM is prevented by cognate chaperone binding (Krampen et al. 2018), TME targeting to the T4SS may be assisted by chaperones.

#### Translocation of T4SS membrane effectors

After being targeted to the recognition site of the T4SS, effector proteins need to be translocated across both bacterial membranes into the host cell. Until now, not much is known about the translocation route for effector proteins in T4ASSs and T4BSSs. Especially the handover of the effector proteins after recognition by the T4CC or, more specifically, the T4CP to the CC is still very unclear because neither DotL nor VirD4 was structurally resolved in complex with the CC structure. Two translocation routes have been proposed (Kitao et al. 2022, Lockwood et al. 2022, Costa et al. 2024). The first one entails the transport of effector proteins across the inner bacterial membrane by the T4CP hexameric channel and further translocation from the periplasm across the outer bacterial membrane by the CC. This is supported by the observed interaction of DotL with effector proteins (Meir et al. 2020). Along the second translocation route, effector proteins are exported in one step across all three membranes through one consecutive translocation channel formed by the CC. According to this model, effectors are handed over by the T4CP/T4CC to the ATPase complex VirB4/DotO and VirB11/DotB, which forms the entry of the cytoplasmic translocation channel (Chetrit et al. 2018, Li et al. 2019, Park et al. 2020, Dutka et al. 2026). This makes it very likely that the substrates, soluble and TMD-containing effectors, enter the translocation channel from the cytoplasmic side. TMEs with high hydrophobicity are proposed to be inserted first into the IM and then extracted again for translocation into the host cell (Malmsheimer et al. 2024). Extraction from the membrane toward the periplasmic or cytoplasmic side comes with a high energy cost, which could be provided by the ATPase VirD4/DotL. Based on the relevance of the cytoplasmic C-terminal secretion signal (Fig. 3b) and the small periplasmic loop (Fig. 3d), the extraction toward the cytoplasm seems most likely. In *L. pneumophila*, larger periplasmic domains in T4B-secreted TMEs have been shown to hinder translocation, likely by interfering with extraction from the IM toward the cytoplasm (Malmsheimer et al. 2024). These features are present not only in *L. pneumophila* TMEs but also in *C. burnetti* and *B. abortus* (Fig. 3c and e), which supports the hypothesis that the two-step secretion pathway is a general principle for target discrimination of TMEs. The access of the translocation channel could also be reached through a lateral approach in the plane of the membrane, but since several TMDs are located around the CC translocation channel this seems rather unlikely, especially for the T4BSS machinery. On the other hand, a lateral approach is utilized by the T4ASS pilus subunit VirB2, which harbors two TMS and a small periplasmic loop (Paiva et al. 1992, Kerr and Christie 2010, Malmsheimer et al. 2024). VirB2, which features a N_out_C_out_ topology (unlike most TMEs), is extracted from the IM toward the periplasm, together with one phospholipid, and is directly recruited to the proposed assembly platform VirB6 (Costa et al. 2016, 2024, Macé et al. 2022a). Since several solutions seem in principle feasible, the exact pathway of engagement of TMEs with the T4CC and the mechanism of membrane extraction remain to be investigated.

That effectors are translocated through the ATPase complex channel is supported by the finding that upon binding to DotB, DotO undergoes a conformational change and opens up a central channel through the ATPase complex and the cylinder (Park et al. 2020). The cylinder is connected to the components of the PR, which ends in the domed OMC with the formation of a pore in the OM by the cage-forming protein DotG (Durie et al. 2020, Macé et al. 2022b, Yue et al. 2025). The translocation channel itself is formed by DotA and IcmX (Yue et al. 2025). Because of its narrow width, substrates of the T4SS need to be at least partially unfolded and it has been shown that folding prevents protein translocation (Amyot et al. 2013, Trokter and Waksman 2018). For T4ASS TMEs, translocation could take place through the pilus structure. While this mechanism has not yet been described, it could resemble a consecutive translocation channel with connection to the host membrane. The structure of the R388 T4ASS showed no consecutive translocation channel, because the stalk blocked the conduit (Macé et al. 2022a). However, the observed structure most likely represents the state of pilus biogenesis and not the actively secreting state.

### Mimicking the ER: the PCV as a specialized site for membrane effector integration

Upon translocation into the host cell, many TMEs are directly inserted into the membrane of the PCV, where they contribute to its formation and remodeling. The PCV membrane represents a specialized interface that frequently adopts structural and compositional features of specific host organelle membranes. In intracellular pathogens employing T4BSSs, such as *L. pneumophila* and *C. burnetii*, as well as in some T3SS-harboring pathogens like *Chlamydia* spp., the respective PCV is tethered to the ER via membrane contact sites (Vormittag et al. 2023, Tsai et al. 2019, Justis et al. 2017, Derré 2015). *Legionella pneumophila* was even shown to transform its LCV into an ER-like compartment by triggering its fusion with ER-derived vesicles, and acquiring rough ER-like features including ribosome association (Tilney et al. 2001, Robinson and Roy 2006). Similarly, in *B. abortus* (T4ASS), PCVs can further develop into interconnected compartments that are continuous with ER cisternae (Sedzicki et al. 2018). Notably, even intracellular pathogens that do not reside within a vacuole, such as *Rickettsia* spp. (T4ASS), form membrane contact sites with the rough ER through their bacterial outer membrane (Acevedo-Sánchez et al. 2023).

The ER is a key target for subversion due to its central role in membrane protein biogenesis, and trafficking of proteins, lipids, and carbohydrates. By remodeling the PCV to resemble ER membranes, pathogens gain access to host biosynthetic pathways, evade immune detection, and establish a stable replicative niche (Roy et al. 2006, Tsai et al. 2019). The remodeling of the PCV into an ER-like compartment is mediated by the secretion of a variety of effectors, which manipulate ER-associated host factors such as Rab GTPases and SNARE proteins to recruit and reorganize the ER around the PCV (Escoll et al. 2016, Tsai et al. 2019, Lockwood et al. 2022).

Importantly, nearly all characterized T4BSS-secreted TMEs from *L. pneumophila* and *C. burnetii* are integrated into the PCV membrane during infection (Table 1). However, when expressed ectopically in the absence of infection, these TMEs predominantly localize to the ER, as observed also for T4ASS-secreted TMEs from *B. abortus* (Table 1). In contrast, infection-coupled ectopic expression restores targeting and insertion into the PCV membrane, as demonstrated for *L. pneumophila* MavE and *C. burnetii* CvpC, CvpD, and CvpE (Vaughn et al. 2021, Larson et al. 2015; Table 1). These findings indicate that the PCV membrane represents the physiological target membrane for these TMEs and suggest a potential role of the ER in facilitating TME insertion into the PCV membrane. In this regard, it is worth mentioning that, while, *L. pneumophila* and *B. abortus* replicate in ER-like vacuoles, *C. burnetii* resides in a phagolysosome-derived compartment (Voth and Heinzen 2007, Vormittag et al. 2023). As phagolysosomal membranes lack the canonical eukaryotic machinery for membrane protein insertion, TME integration into the *Coxiella*-containing vacuole may only rely on ER membrane contact sites or spontaneous membrane insertion during PCV remodeling (Justis et al. 2017).

The ER-like nature of *L. pneumophila* and *B. abortus* PCVs could provide direct access to host factors involved in membrane protein biogenesis. Supporting this hypothesis, proteomic analysis of LCVs isolated from *L. pneumophila*-infected RAW 264.7 macrophages revealed a strong enrichment of ER (16%) and ribosomal (8%) proteins, as well as mitochondrial (25%) and vesicular trafficking-associated (10%) proteins (Hoffmann et al. 2014). Importantly, the LCV proteome contained key components directly involved in membrane protein biogenesis, which may be exploited to promote TME insertion, as discussed in more detail below.

### Strategies for integration of T4BSS-secreted TMEs into host membranes

**Figure 6:**
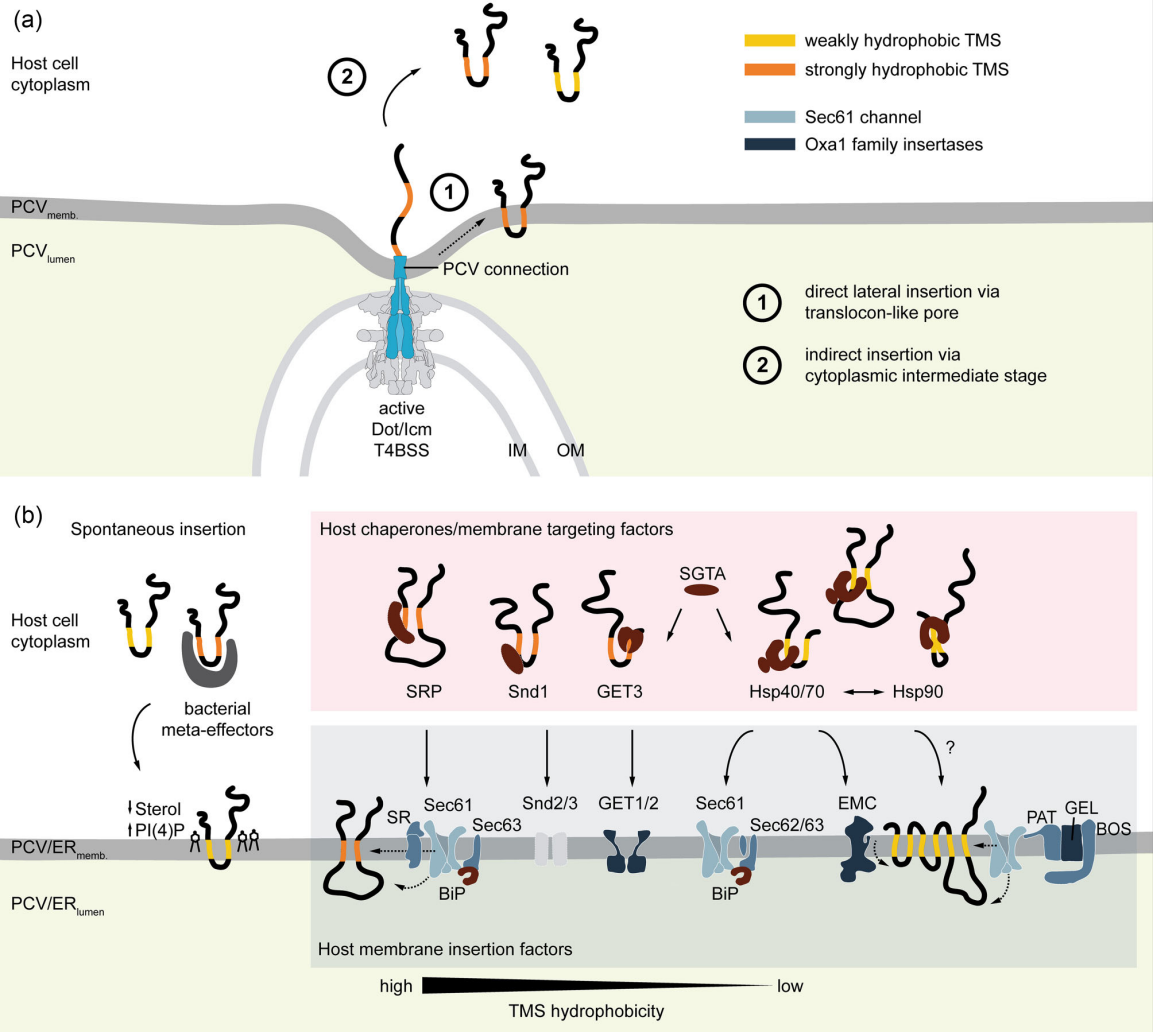
Proposed pathways for host membrane integration of T4BSS**-**translocated TMEs. (a) T4BSS**-**secreted TMEs may integrate into the PCV membrane via two main routes: (1) direct lateral insertion through a translocon**-**like pore formed by the active Dot/Icm T4BSS, or (2) indirect insertion by passing through a cytoplasmic intermediate stage prior to PCV or ER membrane insertion. (b) In the indirect route, TMEs with weakly hydrophobic TMSs (yellow) may remain soluble and integrate spontaneously, potentially aided by low membrane sterol content. Strongly hydrophobic TMSs (orange) may require bacterial cofactors (e.g. meta**-**effectors) or host chaperones for stabilization and delivery to membrane insertion machinery. Depending on TMS hydrophobicity and topology, TMEs may utilize distinct eukaryotic posttranslational targeting and insertion pathways (see Fig. 6). Targeting factors or chaperones (red) are associated with either the Sec61 translocon complex (light blue) or Oxa1 family insertases (dark blue). Strongly hydrophobic TMEs are recognized by the SRP and directed to the SRP receptor (SR) at the ER membrane, enabling lateral integration via Sec61 and translocation of luminal domains with the assistance of Sec63 or Sec62/63 and the luminal chaperone BiP. The less**-**described SRP-independent Snd pathway (Snd1–Snd2/3) accommodates substrates with centrally positioned TMDs. Tail-anchored (TA) TMEs with highly hydrophobic C-terminal TMSs are guided by SGTA and the guided-entry of TA protein (GET) targeting factor 3 to the GET1/2 complex for insertion. Weakly hydrophobic TMEs with variable topology may be chaperoned by Hsp40/70 or Hsp90 (for partially folded proteins), targeting either Sec61 or the ER membrane complex (EMC), depending on the size of luminal loops. While no defined pathway exists for posttranslational insertion of multipass membrane proteins, integration may be mediated by the EMC alone or in coordination with Sec61. Additional folding may involve complexes such as the GEL (GET–EMC-like complex), PAT (intramembrane chaperone), and BOS (Back of Sec61). Abbreviations: IM, inner membrane; OM, outer membrane; PI(4)P, phosphatidylinositol-4-phosphate.

The mechanisms by which *Legionella* TMEs are correctly targeted and inserted into host membranes following secretion via the T4BSS remain largely unresolved. Two conceptual models have been proposed to explain the integration of bacterial TMEs into host membranes (Krampen et al. 2018, Godlee and Holden 2023): one suggests that TMEs are integrated directly into the membrane of the PCV via a translocon-like pore formed by the secretion system, while the other suggests translocation of the TMEs into the host cytoplasm prior to membrane integration (Fig. 6a).

The first hypothesis (Fig. 6a) is supported by the observation that many characterized TMEs are inserted into the membrane that is directly contacted by the T4BSS (Table 1). The process of lateral insertion through a translocon may resemble the lateral gate mechanism of the SecY/Sec61 translocon, which transiently opens to expose the lipid bilayer and facilitate the insertion of hydrophobic TMSs (Berg et al. 2004, Guna and Hegde 2018). While the T3SS is known to employ translocon proteins that oligomerize within host membranes to form such pores (Ide et al. 2001, Veenendaal et al. 2007), definitive structural evidence for a homologous membrane-integrated translocon component within the T4BSS is currently lacking (Dutka et al. 2026, Yue et al. 2025). Nonetheless, certain T4BSS components may contribute to pore formation. Cryo-electron microscopy revealed distinct contact sites between the T4BSS at the bacterial pole and the LCV membrane, implying direct membrane tethering of distal T4BSS components (Böck et al. 2021, Dutka et al. 2026).

Consistent with this, recent structural analyses of actively secreting T4BSS revealed that the DotA–IcmX central protochannel undergoes extensive conformational rearrangements during translocation, forming an extended transenvelope conduit that spans the bacterial cell envelope (Yue et al. 2025). A distinct 15 nm extracellular protrusion emerges upon activation, however, conclusive evidence demonstrating that this structure acts as a functional translocon is still lacking. Moreover, the ability of TMEs to integrate into host membranes when ectopically expressed in eukaryotic cells (Table 1) suggests that translocon-mediated insertion via the T4BSS is not strictly required.

The second model positing a cytoplasmic intermediate stage before membrane insertion is currently favored (Fig. 6a and b). Many TMEs of T4BSS exhibit moderate hydrophobicity (Fig. 6a and b) or possess a highly polar C-terminus (Malmsheimer et al. 2024), biophysical features that may enhance solubility in the host cytosol and promote spontaneous insertion into lipid bilayers (Wimley and White 2000). Additionally, their generally simple architecture, often consisting of only one or two TMS (Fig. 2a–d) and short interconnecting loops (Fig. 6d and e), may facilitate spontaneous membrane insertion (Engelman and Steitz 1981; Fig. 6b). Membrane lipid composition may also influence protein–membrane interactions, including spontaneous insertion into the bilayer and selective binding to specific lipid species (Race et al. 2006). The LCV membrane undergoes dynamic remodeling marked by high lipid turnover and rapid sterol depletion (Vormittag et al. 2023), leading to reduced membrane thickness and increased fluidity, which are properties that may promote the spontaneous insertion of TMEs (Yang et al. 2016).

**Figure 7:**
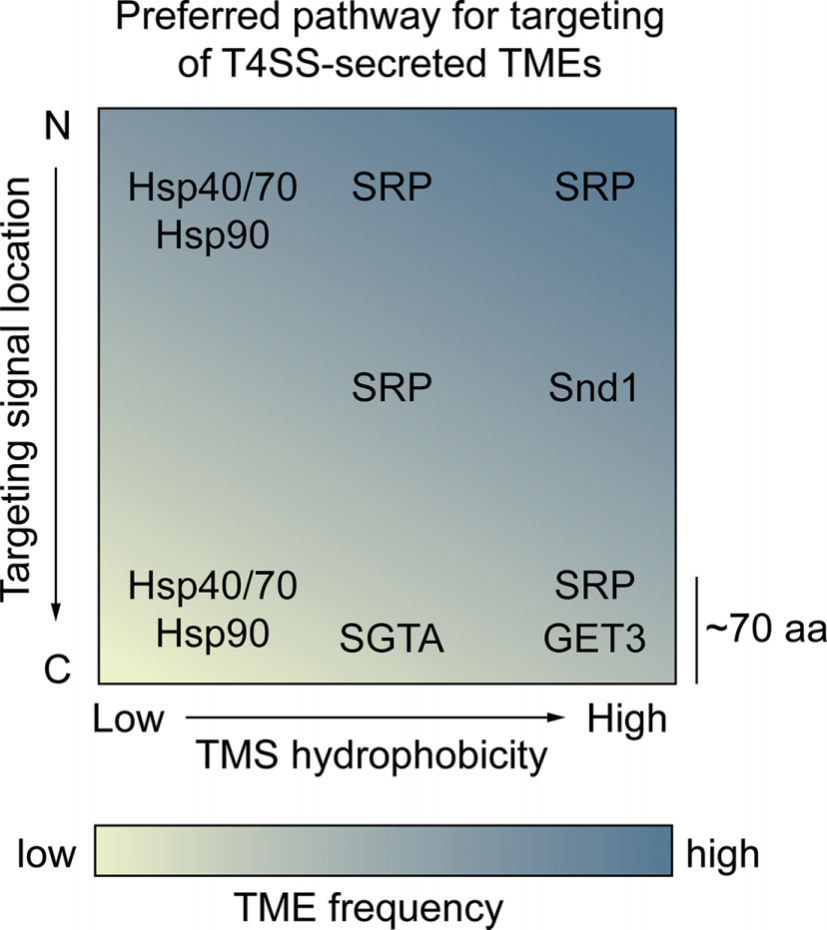
Proposed posttranslational host targeting pathways for T4ASS**-** and T4BSS**-**secreted TMEs to the ER membrane. The position and hydrophobicity of the TMS determine the pathway by which a membrane protein is targeted to the ER membrane. Based on established recognition principles of eukaryotic targeting factors, the frequency of specific biophysical features of T4SS-secreted TMEs was mapped onto characterized ER-targeting pathways (high frequency, dark blue; low frequency, light yellow; see Figs 1–3). Targeting factors include the SRP, SRP-independent targeting factor 1 (Snd1), the chaperones Hsp40, Hsp70, and Hsp90, the chaperone SGTA, and the guided-entry of tail-anchored proteins factor 3 (GET3). Tail-anchored proteins are defined by a TMS located within the C-terminal 65–70 aa.

For TMEs with higher hydrophobicity (Fig. 6a and b), independent membrane targeting and integration are likely inefficient due to an increased risk of aggregation or misinsertion. Consequently, the cytoplasmic intermediate pathway may rely on interactions with chaperones to ensure proper stabilization within the host cytoplasm (Fig. 6b). In this context, *Legionella* could either hijack host chaperones or cosecrete bacterial factors that function as TME-specific chaperones. While the known T4BSS-associated chaperones, IcmSW and LvgA, function primarily as adaptors for effector recognition at the coupling complex and are not secreted (Kwak et al. 2017, Meir et al. 2020), *Legionella* may cosecrete specialized meta-effectors with potential chaperone activity that could bind to and shield the hydrophobic TMD of the TMEs from the host cytoplasm. Although several meta-effectors have been shown to modulate effector activity within the host, direct evidence for their role in stabilizing TMEs or guiding TME membrane targeting is still lacking (Joseph and Shames 2021, Mount et al. 2024). Alternatively, T4BSS-secreted TMEs may hijack host chaperone systems, which are evolutionarily conserved and highly versatile in targeting a broad range of substrates (Fig. 6b). Recruitment of these chaperones is often influenced by the hydrophobicity and position of the TMSs (Taipale et al. 2012, Bogumil et al. 2014), making them well-suited to accommodate the structural diversity of T4BSS-secreted TMEs (Fig. 7). The integration of TMEs into host cell membranes may further depend on host cell factors such as receptors for membrane-targeting and insertases or translocases for membrane insertion (Fig. 6b). The ER-like characteristics of the LCV membrane, including the presence of host membrane protein insertion machinery provide an optimal environment for the guided insertion of T4BSS-secreted TMEs into their functional target membrane, predominantly the LCV, supporting the cytoplasmic intermediate model of TME membrane integration.

### Host membrane protein biogenesis as a gateway for TME insertion

In eukaryotic cells, almost all membrane proteins are initially inserted into the ER membrane, where they are processed and sorted to their final destinations. As the ER membrane evolved from the prokaryotic plasma membrane, the core machineries of membrane protein biogenesis are conserved and mechanistically related across domains of life (Baum and Baum 2014, Hegde and Keenan 2022). This evolutionary relationship suggests that bacterial TMEs may exploit host targeting and insertion pathways following T4SS secretion, aligning with the proposed model in which TMEs transit through a cytoplasmic intermediate before membrane integration (Fig. 6a and b). While the route(s) T4SS-secreted TMEs take from the delivery into the host cytosol to the target membrane remain(s) to be fully elucidated, they can be potentially reconstructed using mechanistic insights from eukaryotic membrane protein biogenesis.

#### Targeting of T4SS-secreted TMEs to ER membranes

Membrane protein biogenesis universally involves two key steps: targeting, whereby the hydrophobic TMS is recognized and delivered to the membrane, and insertion, whereby TMSs are integrated into the lipid bilayer along with translocation of flanking hydrophilic regions. These two steps can occur co- or posttranslationally, depending largely on the position and hydrophobicity of the targeting sequence (Fig. 7). Typically, the most N-terminal hydrophobic segment, either a cleavable SP or the first TMS, serves as the targeting signal. SPs, which are located at the extreme N-terminus, have a characteristic tripartite structure consisting of a basic N-domain, a 7–13 aa hydrophobic H-domain, and a polar C-domain (Hegde and Bernstein 2006, Liaci and Förster 2021). If the targeting signal lies within ∼65–70 residues from the C-terminus, cotranslational targeting by the SRP becomes less likely, as translation may terminate before the ribosome reaches the ER. This distance constraint reflects the length of the nascent peptide within the ribosome exit tunnel and the number of residues that can still be incorporated into the nascent polypeptide before ER targeting is completed. Consequently, these tail-anchored (TA) membrane proteins (Fig. 3a) typically rely on posttranslational targeting pathways (Hegde and Keenan 2022). Structural predictions using DeepTMHMM indicate that 5%–30% of T4A- and T4B-secreted TMEs possess N-terminal SPs (Fig. 6a and b). However, as TME translation occurs in the bacterial cytosol, these SPs are likely cleaved by bacterial signal peptidases prior to translocation via the T4SS (Luirink et al. 2012). Consequently, the first hydrophobic TMS emerging from the T4SS would serve as the functional signal for membrane targeting within the host. Posttranslational targeting and insertion pathways are probably favored by bacterial TMEs, as they offer a broader and more flexible repertoire of targeting factors and translocation complexes. These pathways are also better suited to accommodate the high throughput of effector protein secretion, in contrast to the slower kinetics of cotranslational translocation, constrained by the eukaryotic ribosomal elongation rate of only ∼8 aa per second (Mathews et al. 2000). Given the diversity in targeting signal position and biophysical characteristics among T4A- and T4B-secreted TMEs (Figs 1–4), a single host membrane targeting pathway is unlikely to suffice for all effector types (Figs 6b and 7).

For delivering highly or moderately hydrophobic membrane proteins to the ER, the SRP pathway is the primary and best-characterized mechanism. Upon binding to TMSs or SPs on nascent polypeptides, SRP temporarily halts translation and guides the ribosome–nascent chain complex to the ER membrane, where it is received by the SRP receptor (SR) and Sec61 translocon for membrane insertion (Liaci and Förster 2021). Although the SRP pathway predominantly supports cotranslational targeting, which would be incompatible with bacterial TMEs, it can also mediate posttranslational delivery to the Sec61 translocon, albeit with reduced efficiency due to its higher affinity for actively translating ribosomes (Abell et al. 2007, Johnson et al. 2013). Notably, intracellular pathogens such as *L. pneumophila* secrete effectors that can block host protein biosynthesis to scavenge for aas (Belyi 2020, Lockwood et al. 2022, Subramanian et al. 2023). This translational block may also lead to an increase in the availability of unbound SRP, thereby promoting SRP-dependent posttranslational targeting of bacterial TMEs (Fig. 7).

Highly hydrophobic TA membrane proteins, characterized by a TMS within 65–70 aa of the C-terminus, are typically targeted and inserted posttranslationally via the guided entry of TA proteins (GET) pathway. Substrate loading onto the targeting factor GET3 is mediated by a pretargeting complex comprising SGTA and the Bat3 complex, both of which associate with the ribosome exit site (Mariappan et al. 2010, Hegde and Keenan 2022). However, SGTA can also capture TMD substrates that were already released into the cytosol or transferred from Hsp70 chaperones (Cho and Shan 2018). This cascade selectively delivers highly hydrophobic TMDs to GET3 for insertion via the ER-resident GET1–GET2 complex. In contrast, less hydrophobic TMDs remain associated with SGTA or are rerouted to alternative posttranslational targeting pathways.

For membrane proteins with moderate or low hydrophobicity, i.e. poor substrates for SRP or GET3, the Hsp70/Hsp40 chaperone system facilitates posttranslational targeting to either the Sec61 translocon with Sec62/63 auxiliary components or the ER membrane complex (EMC). These chaperone networks provide greater substrate flexibility, accommodating a wide range of TMD topologies, hydrophobicities, and folding intermediates, making them well-suited for processing T4SS-secreted TMEs (Liaci and Förster 2021). Hsp40 functions as a cofactor of Hsp70, enhancing ATP-dependent cycling of Hsp70 between binding and release states, thereby shielding the hydrophobic regions and preventing premature folding or aggregation of the TMD substrate (Frydman and Höhfeld 1997). Downstream of Hsp70, Hsp90 may assist in recognizing partially folded TMD-containing intermediates (Luengo et al. 2019).

An additional targeting route for T4SS-secreted TMEs could be the SRP-independent (Snd) pathway, which serves as a compensatory mechanism for substrates not efficiently recognized by the SRP or GET systems. The Snd pathway accommodates a broad range of proteins, particularly those with centrally located TMDs of variable hydrophobicity (Aviram et al. 2016). While Snd1 functions as a soluble cytosolic targeting factor that interacts with ribosomes, the ER membrane receptor hSnd2/Snd3 mediates substrate transfer to the Sec61 translocon for membrane insertion (Haßdenteufel et al. 2017).

The diversity in TMD position and hydrophobicity among T4A- and T4B-secreted TMEs implies that different posttranslational targeting pathways may be utilized inside the host (Fig. 7). The predominance of moderately hydrophobic TMEs (Fig. 6a and b) suggests primary engagement with the Hsp70/40 system, or Hsp90 depending on folding status. In contrast, highly hydrophobic TMEs with N-terminal or central TMDs may be recognized by free SRP or the less abundant Snd1 pathway, while TA proteins bearing C-terminal TMDs are most likely targeted via the GET pathway (Fig. 7).

#### Insertion of T4SS-secreted TMEs into ER membranes

Upon delivery to the host membrane, the TMSs of T4SS-secreted TMEs need to be integrated into the lipid bilayer. This insertion is facilitated by one or both of the two universally conserved membrane protein translocation systems: the Sec61 complex (homologous to prokaryotic SecY) and members of the Oxa1 superfamily (Fig. 6b). The Oxa1 superfamily of membrane protein insertases, including bacterial YidC, mitochondrial Oxa1, and the ER-resident proteins EMC3, GET1, and TMCO1, are evolutionarily related and share a core mechanism for TMS insertion involving a hydrophilic vestibule to aid flanking domain translocation. However, due to the absence of a membrane-spanning channel, this translocation process is limited to less than ∼50 aa (Kizmaz and Herrmann 2023, Hegde and Keenan 2024). In addition to inserting single tail-anchored TMSs, Oxa1 family members can also translocate short hydrophilic loops between adjacent TMSs, which are likely translocated through the vestibule concomitantly with the insertion of neighboring TMSs, either in rapid succession or as a hairpin. This makes Oxa1 family insertases a favorable insertion factor for most T4SS-secreted TMEs, since they typically adopt an N_in_C_in_ topology with two TMS and short interconnecting loops (Fig. 3a–e). Interestingly, in prokaryotes the Oxa1 insertase YidC is more abundant than the SecY translocon (Drew et al. 2003), whereas the opposite is true for the respective eukaryotic homologs (Smalinskaitė and Hegde 2023). This distribution suggests that Oxa1-mediated membrane insertion is optimized for organisms with fast growth rates and less complex membrane proteomes, likely due to its capacity for a more rapid membrane integration compared to the slower translocation rates of the Sec61 translocon. Consequently, eukaryotic Oxa1 family insertases at the ER, such as the EMC or GET complex, would be able to accommodate a high load of bacterial TMEs with simple N_in_C_in_ topologies that are injected into the host cell by the T4SS during infection.

Insertion by Oxa1 family insertases through a hydrophilic vestibule may not suffice for T4SS-secreted TMEs with a higher TMS complexity (Fig. 2a–d) and those with long luminal-facing C-tails (Fig. 6f and g). Unlike Oxa1 insertases, the Sec61 translocon contains an aqueous channel that can accommodate the translocation of long, unfolded, or loosely folded polypeptide segments (Rapoport et al. 2017). To drive unidirectional passage through this passive pore, Sec61 cooperates with accessory complexes that provide the necessary directional force. Cotranslational translocation via the Sec61 channel is driven by the slow elongation speed of the ribosome–nascent chain complex. The transfer of the translating ribosome to Sec61 enables the hydrophobic signal sequence to engage the lateral gate, displacing the central plug and initiating translocation (Hegde and Keenan 2022).

For posttranslational translocation, the Sec61 channel cooperates with the Sec62/63 complex and the luminal Hsp70 ATPase BiP, which functions as a molecular ratchet to drive polypeptide translocation (Rapoport et al. 2017, Liaci and Förster 2021). Sec63 is central to this process, engaging Sec61 to partially open the channel gate and recruiting BiP from the ER lumen, as well as serving as a docking site for the Sec71/72 subcomplexes that recruit Hsp70 targeting factors from the cytosol. Sec62 further facilitates full plug displacement, enabling efficient posttranslational translocation (Johnson et al. 2013, Tripathi et al. 2017, Shan 2024). Sec63 also participates in both SRP-dependent and -independent pathways, as the SR can compete with Sec62 for binding to Sec63 and/or Sec61, thereby modulating Sec62 association (Young et al. 2001, Jan et al. 2014, Jadhav et al. 2015). Thus, depending on the TMS hydrophobicity, T4SS-secreted bacterial TMEs with long translocated domains may be posttranslationally targeted by the SRP or Hsp70/Hsp40 systems and inserted into the ER membrane via Sec61, through an SRP-dependent or -independent mechanism (Fig. 6b).

The integration of complex multipass membrane proteins, also found to be secreted by the T4SS (Fig. 2a–d), is typically coordinated cotranslationally by dynamic switching between Sec61 and Oxa1 family insertases. In this iterative model, Oxa1 facilitates insertion of TMSs flanked by short translocated domains, while Sec61 is engaged when longer hydrophilic domains follow the TMS (Hegde and Keenan 2024). Oxa1 family insertases involved in this process are either the EMC (Wu and Hegde 2023, Wu et al. 2024), or the GET-and EMC-like complex (GEL), which forms the multipass translocon together with the intramembrane chaperone (PAT) and the back of Sec61 (BOS) complex (Sundaram et al. 2022). This multipass translocon resembles the prokaryotic holotranslocon, both featuring a SecY-family channel, an Oxa1-type insertase, and a lipid-filled cavity (Hegde and Keenan 2022). Consequently, T4SS-secreted multipass TMEs with diverse topologies may exploit this machinery for membrane integration. However, given that this process is primarily described for cotranslational insertion, these effectors may instead rely entirely on Oxa1-family insertases, with proper membrane topology maintained postinsertion by the PAT chaperoning complex (Fig. 6b). Notably, in membrane proteins with ≥4 TMSs, most luminal loops were found to be shorter than 50 aa in length (mean ∼15 aa; Wallin and Heijne 1998), consistent with the predicted topology of T4SS-secreted multipass TMEs (Fig. 6d and e).

In summary, T4SS-secreted TMEs are unlikely to follow a single linear pathway for membrane targeting and insertion in the host. Instead, their routing is likely diversified into multiple posttranslational pathways at each step, based on intrinsic features, such as TMS hydrophobicity, topology, and domain length (Figs 6 and 7). Given that most TMEs exhibit moderate hydrophobicity and a simple N_in_C_in_ two-TMS topology (Figs 1–4), Oxa1 family insertases, particularly the EMC, are likely favored for rapid membrane integration. Their targeting to the EMC might be facilitated by general chaperones such as Hsp70/40 or Hsp90. In contrast, highly hydrophobic TA proteins are more likely to engage the GET pathway for both targeting and insertion. TMEs with longer hydrophilic domains, which may exceed the translocation capacity of Oxa1 insertases, could instead utilize the Sec61 translocon, either through SRP-dependent or Hsp70-mediated mechanisms. Alternative targeting routes, such as the Snd pathway, may also contribute to efficient targeting and membrane integration of the diverse repertoire of T4SS-secreted TMEs.

## Conclusion and outlook

Membrane effectors take a relevant role in host cell manipulation to ensure T4SS-dependent intracellular survival. Research in recent years has made great progress in elucidating the function of the growing number of membrane effector proteins and their site of activity in the host cell membranes. Moreover, critical aspects of T4SS-dependent effector targeting and translocation have become more clear with the elucidation of the structure of the T4SS translocation machinery in different bacteria. Further integrating knowledge of membrane protein biogenesis in prokaryotes enlightened the possible secretion pathways, especially for hydrophobic TMEs. Nevertheless, the detailed steps of TME handling on the pathogen and host site remain unknown.

The two-step secretion pathway with an IM intermediate seems a central and specialized mechanism for targeting TMEs to the T4SS, however, the precise molecular details and the identity of factors involved remain to be elucidated (Malmsheimer et al. 2024). Also, the mechanisms responsible for recognizing and targeting TMEs with weakly hydrophobic TMSs to the T4SS remain unknown.

The intrinsic capacity of moderately hydrophobic TMEs to insert into host membranes independent of host factors also requires further investigation as does the identification of interacting host proteins that may assist in TME targeting or membrane integration.

## Supplementary Material

uqag007_Supplemental_File
